# Drug-Coated Balloon-Based Versus Drug-Eluting Stent-Only Strategy in Stable Angina and Acute Coronary Syndrome: A Propensity-Score Overlap-Weighted Analysis

**DOI:** 10.3390/jcm15166140

**Published:** 2026-08-07

**Authors:** Yong Hoon Kim, Ae-Young Her, Sunwon Kim, Dong Oh Kang, Chang-Bae Sohn, Eun-Seok Shin

**Affiliations:** 1 Division of Cardiology, Department of Internal Medicine, Kangwon National University College of Medicine, Chuncheon 24341, Republic of Korea; hermartha1@gmail.com; 2 Division of Cardiology, Department of Internal Medicine, Kangwon National University Hospital, Chuncheon 24289, Republic of Korea; 3 Department of Cardiology, Korea University Ansan Hospital, Korea University College of Medicine, Ansan 15355, Republic of Korea; sunwon11@hanmail.net; 4 Cardiovascular Center, Department of Internal Medicine, Korea University Guro Hospital, Korea University College of Medicine, Seoul 08308, Republic of Korea; gelly9@naver.com; 5 Department of Cardiology, Ulsan Medical Center, Ulsan 44686, Republic of Korea; scb77@hanmail.net; 6 Department of Cardiology, Ulsan University Hospital, University of Ulsan College of Medicine, Ulsan 44033, Republic of Korea

**Keywords:** drug-coated balloon, drug-eluting stent, stable angina, acute coronary syndrome, percutaneous coronary intervention

## Abstract

**Background**: Whether the comparative effectiveness of a drug-coated balloon (DCB)-based versus drug-eluting stent (DES)-only percutaneous coronary intervention (PCI) strategy differs between stable angina (SA) and acute coronary syndrome (ACS) remains uncertain. **Methods**: In this observational, registry-based study, we analyzed 11,522 patients (4490 with SA and 7032 with ACS) from two multicenter registry sources who had undergone PCI in routine clinical practice; the treatment strategy was not assigned by a study protocol. Within each presentation, the DCB-based and DES-only strategies were compared using propensity-score overlap weighting. Complementary within-strategy analyses compared SA with ACS. The primary outcome was the 3-year rate of major adverse cardiac and cerebrovascular events (MACCE). **Results**: In both presentations, a DCB-based strategy was associated with a lower 3-year risk of MACCE than a DES-only strategy, with a stronger association in SA (overlap-weighted hazard ratio [HR], 0.33; 95% confidence interval [CI], 0.22–0.49) than in ACS (HR, 0.73; 95% CI, 0.59–0.91; *p*-for-interaction < 0.001). Within-strategy analyses localized this effect modification to the DCB-based group: SA was associated with a lower risk of MACCE than ACS (HR, 0.47; 95% CI, 0.31–0.69) in the DCB-based group, whereas this gradient was absent in the DES-only group. The incidence of major bleeding was lower with the DCB-based strategy in both presentations. **Conclusions**: A DCB-based PCI strategy was associated with lower 3-year event rates than a DES-only strategy in both SA and ACS, with the strongest relative association in SA. These observational findings are hypothesis-generating and warrant confirmation in randomized trials.

## 1. Introduction

Stable angina (SA) generally reflects a stable plaque that causes fixed, flow-limiting stenosis with limited thrombus and local inflammation, whereas acute coronary syndrome (ACS) usually arises from plaque rupture or erosion with a superimposed thrombus, vasoconstriction, and dynamic vessel caliber [1]. A drug-coated balloon (DCB) delivers an antiproliferative agent during a single transient inflation and leaves no permanent implant; unlike a drug-eluting stent (DES), its efficacy may depend strongly on the lesion substrate at the time of treatment, making presentation-dependent effectiveness biologically plausible [2]. Trials and registries have supported the use of DCB-based PCI in de novo coronary disease, particularly small-vessel or stable presentations [3,4] and ACS [5,6]; however, direct comparisons between presentations are limited. The most relevant prior analysis, from Basel Kosten Effektivitäts Trial-Drug-Coated Balloons Versus Drug-Eluting Stents in Small Vessel Interventions (BASKET-SMALL 2), compared acute and chronic presentations in small-vessel disease and found no interaction for the composite endpoint [7]. Thus, whether the DCB-versus-DES treatment effect differs between SA and ACS across a broader PCI population, and within which treatment group any difference originates, remains unresolved. Accordingly, in a large cohort combining two multicenter registries [8,9], we compared 3-year rates of major adverse cardiac and cerebrovascular events (MACCE) between DCB-based and DES-only strategies within the SA and ACS strata by using propensity-score overlap weighting, formally tested treatment-by-presentation interactions, and then compared SA with ACS within each strategy to identify the source of any interaction.

## 2. Methods

### 2.1. Study Population and Registry Sources

**Figure 1:**
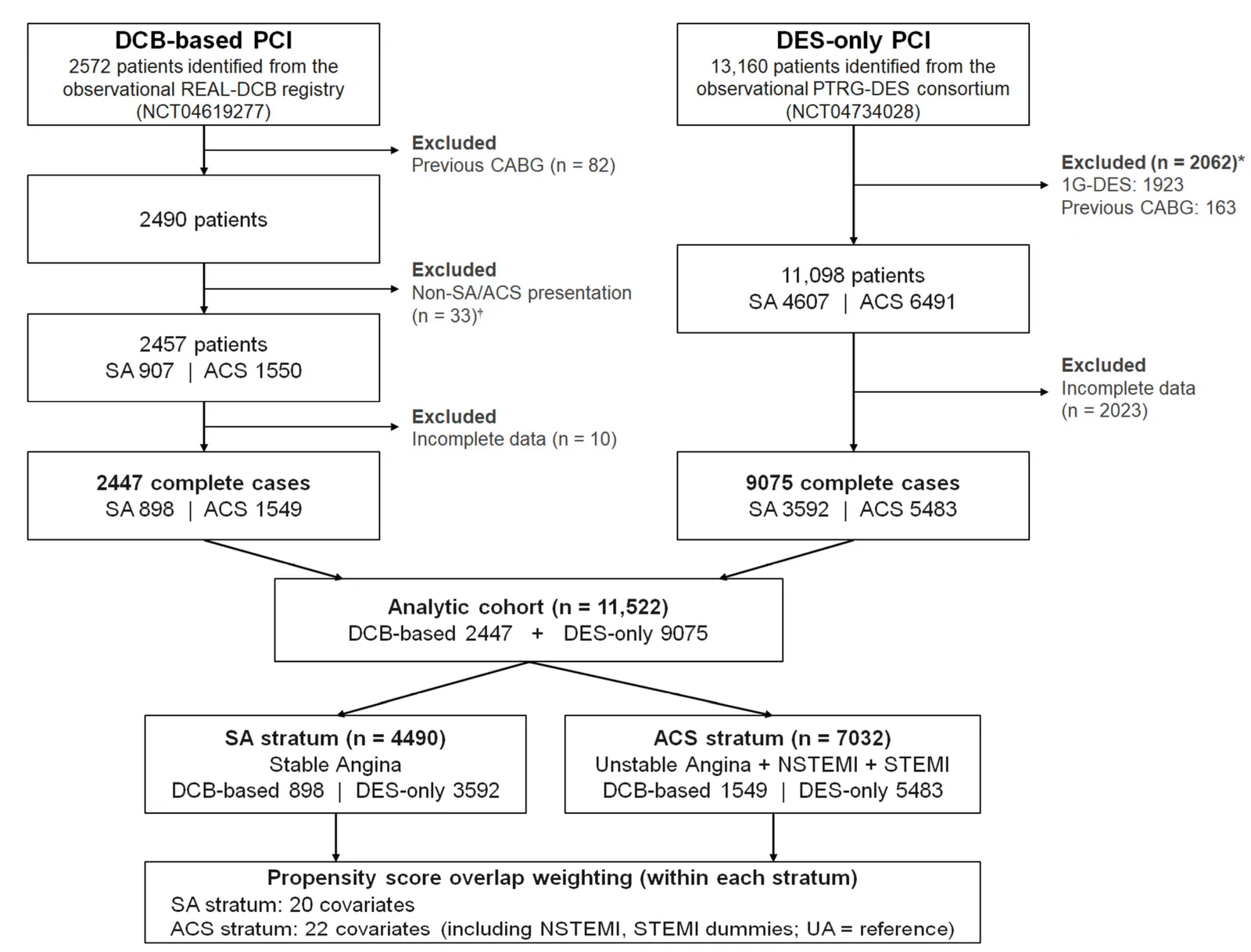
Study flow diagram and analytic cohort selection from two observational, registry-based PCI cohorts. Treatment strategy was not assigned by a study protocol but was determined by the treating physicians according to routine clinical practice. * In the DES cohort, the sum of individual exclusion counts (2086) exceeds the unique number of patients excluded (2062) because 24 patients had both first-generation DES and previous CABG. ^†^ Non-SA/ACS presentations included asymptomatic ischemia, other presentations, and aborted sudden cardiac death. ACS indicates acute coronary syndrome; CABG, coronary artery bypass grafting; DCB, drug-coated balloon; DES, drug-eluting stent; 1G-DES, first-generation drug-eluting stent; NSTEMI, non-ST-segment elevation myocardial infarction; SA, stable angina; STEMI, ST-segment elevation myocardial infarction; and UA, unstable angina.

This was an observational, registry-based analysis conducted in a stratified multicenter Korean cohort assembled from complementary PCI registries representing the two treatment strategies. The PCI device strategy was not assigned by a study protocol, and no patients were prospectively allocated to a DCB-based or DES-only strategy for the purpose of this analysis. All PCI procedures, device selections, and antithrombotic decisions were made by the treating physicians according to routine clinical practice and local institutional standards. The registry identifiers are provided for transparency regarding the source registries and should not be interpreted as registration of a randomized treatment trial for the present analysis. Patients treated with a DCB-based strategy were identified from the REAL-DCB registry (Impact of Drug-Coated Balloon Treatment in De Novo Coronary Lesion; NCT04619277) [8] and an investigator-initiated dataset compiled across five tertiary teaching hospitals in Korea. Patients treated with a DES-only strategy were drawn from the Platelet Function and Genotype-Related Long-Term Prognosis in Drug-Eluting Stent-Treated Patients with Coronary Artery Disease (PTRG-DES) consortium (NCT04734028) [9], a nationwide collaborative network of 32 academic centers that contributed data on 13,160 patients treated with DES between July 2003 and August 2018, of whom recipients of second-generation devices were eligible for the present analysis. Eligible patients underwent successful PCI between November 2003 and December 2023. Patients were excluded if they had undergone prior coronary artery bypass surgery, presented with cardiogenic shock at the index admission, received preprocedural fibrinolytic therapy, showed angiographic failure of the culprit-lesion intervention, or had no postprocedural follow-up data. The clinical presentation at the index PCI was categorized prospectively as SA, unstable angina, non-ST-segment elevation myocardial infarction (NSTEMI), or ST-segment elevation myocardial infarction (STEMI). The analytical cohort consisted of 11,522 patients: 4490 with SA (DCB-based, 898; DES-only, 3592) and 7032 with ACS (DCB-based, 1549; DES-only, 5483) (Figure 1). Institutional Review Board approval was obtained from each participating site, and the requirement for written informed consent was waived because the study was conducted using de-identified registry data.

Registry registration: Impact of Drug-Coated Balloon Treatment in De Novo Coronary Lesion; ClinicalTrials.gov Identifier: NCT04619277; URL: https://clinicaltrials.gov/study/NCT04619277 (accessed on 4 August 2026). Registry registration: PTRG-DES Consortium (PTRG); ClinicalTrials.gov Identifier: NCT04734028; URL: https://clinicaltrials.gov/study/NCT04734028 (accessed on 4 August 2026).

### 2.2. Procedural Protocol and Antithrombotic Management

In the DCB-based strategy, the procedural sequence followed contemporary consensus statements for coronary DCB use [10,11]. Adequate lesion preparation was required before DCB inflation and was defined as residual diameter stenosis no greater than 30%, Thrombolysis in Myocardial Infarction (TIMI) grade 3 antegrade flow, and no flow-limiting dissection. Once these criteria were met, a paclitaxel-iopromide-coated balloon (SeQuent Please; B. Braun, Melsungen, Germany) was inflated at the target site. Lesions that could not be adequately prepared underwent DES implantation, and patients requiring rescue stenting within a DCB-treated segment were excluded from the analytic dataset. Eligibility was therefore not symmetric between the groups: entry into the DCB-based group was conditional on an acceptable acute result after lesion preparation, whereas no comparable procedural filter was applied to the DES-only group. Because the registry recorded only those patients in whom a drug-coated balloon was ultimately delivered, the number whose lesion preparation failed or who required bailout stenting cannot be recovered. Because device selection was made lesion-by-lesion, the DCB-based group included DCB-only PCI and hybrid PCI, in which a DCB was used at one lesion or segment and a DES at another. After PCI, DAPT duration was guided by both presentation and treatment strategy: 1 to 3 months for stable patients undergoing DCB-only PCI, 6 months for stable patients receiving DES, and 12 months for patients with ACS regardless of device type. In hybrid DCB–DES procedures, the DES-based DAPT recommendation was applied [10,11].

### 2.3. Endpoints and Follow-Up

The primary endpoint was the 3-year rate of MACCE, defined a priori as cardiac death, MI, stroke, or target-vessel revascularization (TVR). The secondary endpoints were individual MACCE components, target-lesion or stent thrombosis (TL/ST), major bleeding, and net adverse clinical events (NACE), defined as MACCE plus major bleeding. Cardiac death was defined as any death without a clearly demonstrable noncardiac cause [12]. MI was adjudicated according to the Fourth Universal Definition of Myocardial Infarction [13], and stroke was defined as an acute cerebrovascular event resulting in death, persistent neurological impairment lasting more than 24 h, or imaging evidence of cerebral infarction [14]. TL/ST definitions followed the Academic Research Consortium-2 (ARC-2) framework [12]; TVR included all clinically indicated revascularizations of a target vessel during follow-up; and major bleeding was classified as Bleeding Academic Research Consortium (BARC) type 3 to 5 [15]. Follow-up assessments were performed through predefined outpatient visits supplemented by structured telephone contact when in-person evaluation was not feasible. Clinical events were captured from hospital medical records and discharge documents, with site-level adjudication according to the ARC-2 criteria and a consensus review for disagreements.

### 2.4. Statistical Analysis

Propensity-score overlap weighting was adopted as the principal analytic framework to mitigate measured confounding in this observational, non-randomized comparison, concentrating analytic weight on patients in clinical equipoise between the two strategies [16]. Treatment-assignment probabilities were estimated separately within each stratum by multivariable logistic regression, prioritizing outcome-associated covariates [17,18,19]. The SA model included 20 covariates and the ACS model 22 (adding NSTEMI and STEMI indicators); two device-burden variables were excluded for multicollinearity. The full covariate list, selection rationale, and collinearity diagnostics are provided in the Supplementary Methods and Tables S1–S3. Covariate balance was assessed by absolute standardized mean differences (threshold 0.10; Figure S1). Three-year cumulative event probabilities were estimated by the Kaplan–Meier method on the overlap-weighted cohort, with overlap-weighted Cox proportional-hazards regression using robust variance estimators; an HR below 1.0 indicated a lower event rate in the DCB-based group. Treatment-by-presentation heterogeneity was tested with an interaction term (*p*-for-interaction). To localize any effect modification, SA was compared with ACS within each treatment group separately, with presentation-propensity weights re-estimated within the respective group. Five predefined sensitivity analyses and eight predefined subgroup analyses were performed (Supplementary Methods; Tables S4 and S5; Figure S2). All tests were two-sided (*p* < 0.05); analyses used R 4.3.0 (R Foundation for Statistical Computing, Vienna, Austria).

## 3. Results

### 3.1. Baseline Characteristics

Of the 11,522 patients, 2447 underwent a DCB-based strategy and 9075 underwent DES-only PCI. In the overall cohort, DCB-treated patients were slightly younger and more frequently male, with a heavier burden of cardiovascular risk factors and prior disease and angiographically more complex lesions (Table S6). The same directional differences were evident within each stratum before weighting. In the SA cohort (Table 1), the DCB-based group was more frequently male with higher rates of diabetes mellitus, previous PCI, and previous stroke, whereas age was comparable; multivessel disease, bifurcation lesions, and American College of Cardiology/American Heart Association (ACC/AHA) type B2/C lesions were more frequent. In the ACS cohort (Table 2), the DCB-based group was again more frequently male with higher rates of diabetes mellitus, previous MI, and previous PCI; unstable angina was more common in the DCB-based group, whereas NSTEMI and STEMI were more common in the DES-only group, and lesion complexity was again greater. After overlap weighting, all modeled covariates were balanced (absolute SMD < 0.10). Baseline characteristics for the within-strategy SA-versus-ACS comparisons are provided in Tables S7 and S8.

### 3.2. Clinical Outcomes

#### 3.2.1. DCB-Based Versus DES-Only Strategy by Clinical Presentation

**Figure 2:**
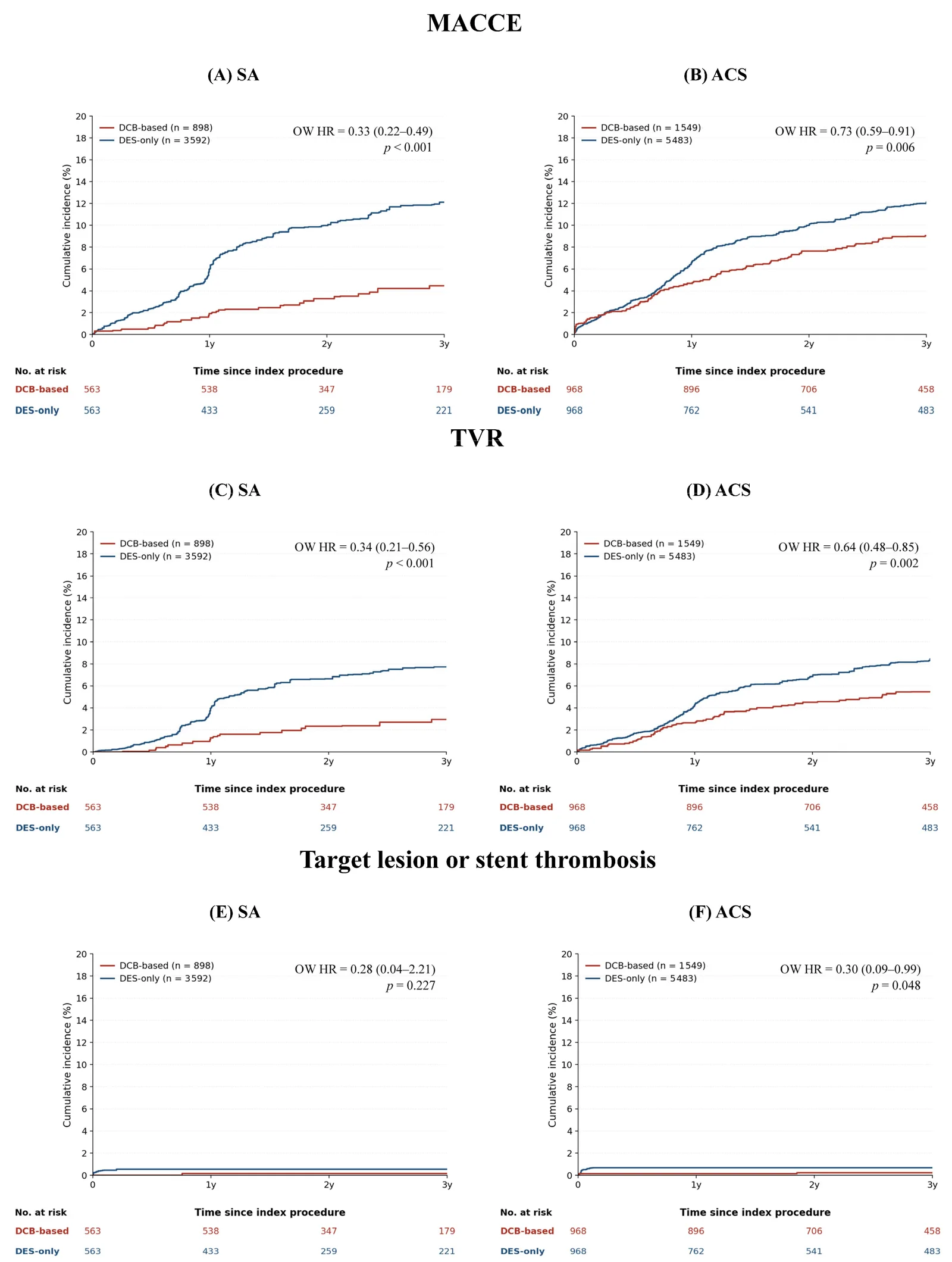

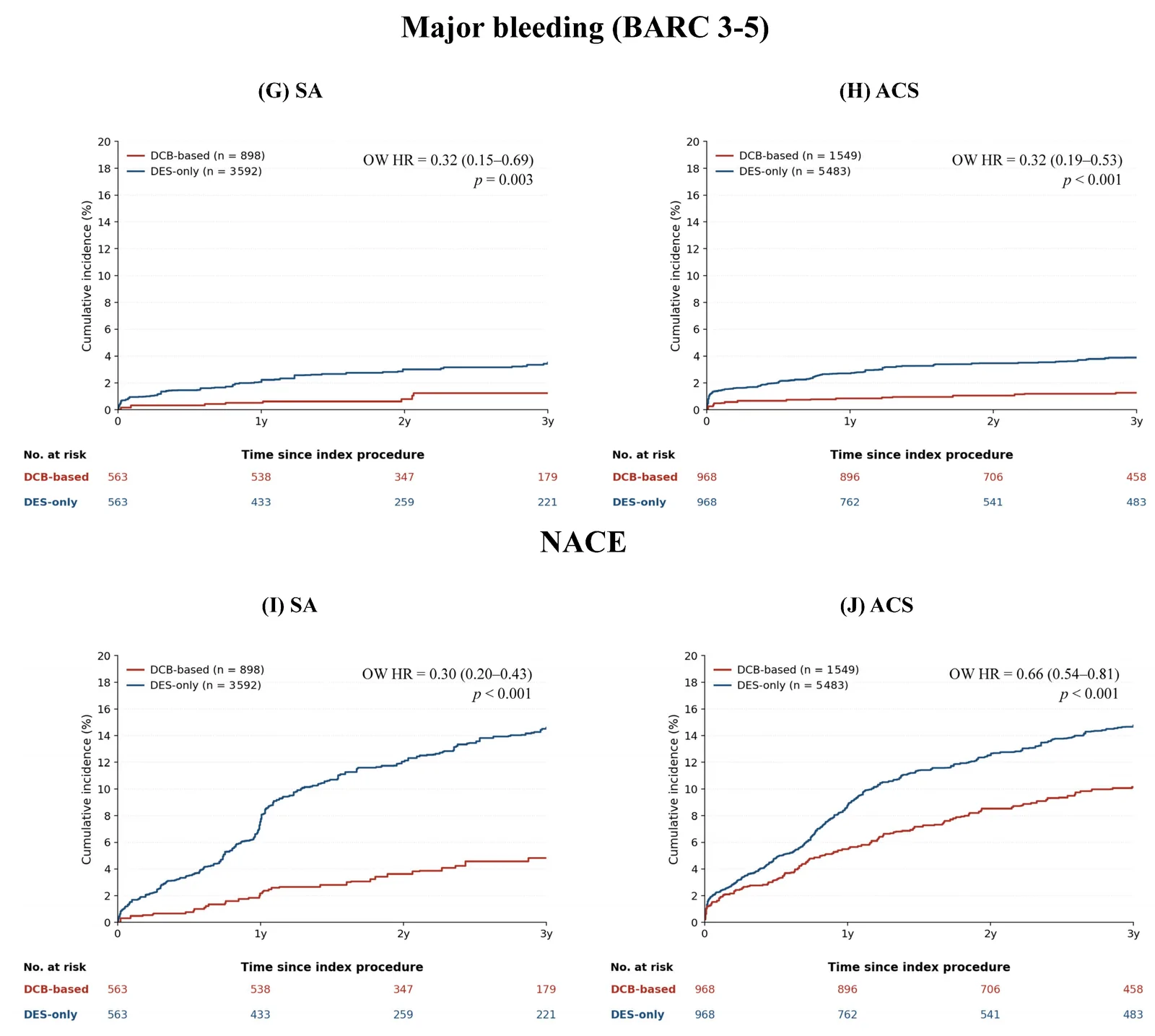
Three-year cumulative incidence of clinical outcomes with significant between-strategy differences in at least one clinical stratum, comparing DCB-based versus DES-only PCI: MACCE (**A**,**B**), TVR (**C**,**D**), TL/ST (**E**,**F**), major bleeding (**G**,**H**), and NACE (**I**,**J**). Each endpoint is shown for the stable angina (SA) stratum (left) and the acute coronary syndrome (ACS) stratum (right). Cumulative incidence was estimated by the Kaplan–Meier method after overlap weighting; overlap-weighted hazard ratios (HRs) with 95% confidence intervals (CIs) are from Cox proportional-hazards models, with an HR below 1 favoring the DCB-based strategy. Numbers below each panel are the overlap-weighted effective patients at risk at yearly intervals. ACS, acute coronary syndrome; CI, confidence interval; DCB, drug-coated balloon; DES, drug-eluting stent; HR, hazard ratio; MACCE, major adverse cardiac and cerebrovascular events; NACE, net adverse clinical events; OW, overlap weighting; SA, stable angina; TL, target lesion; ST, stent thrombosis; TVR, target-vessel revascularization.

The primary endpoint of 3-year MACCE occurred in 33 of 898 patients (3.7%) in the DCB-based group versus 282 of 3592 (7.9%) in the DES-only group in the SA cohort, and in 134 of 1549 (8.7%) versus 463 of 5483 (8.4%) in the ACS cohort. After overlap weighting, a DCB-based strategy was associated with a markedly lower risk of MACCE in SA (HR, 0.33; 95% CI, 0.22–0.49; *p* < 0.001) and a more modest reduction in ACS (HR, 0.73; 95% CI, 0.59–0.91; *p* = 0.006), and the association differed significantly between presentations (*p*-for-interaction < 0.001). Similar patterns were observed for TVR and NACE (Figure 2), each with a greater relative reduction in SA (*p*-for-interaction = 0.037 and <0.001, respectively). Major bleeding was reduced to a similar degree in both presentations (*p*-for-interaction = 0.943). TL/ST reached significance only in ACS (HR, 0.30; 95% CI, 0.09–0.99; *p* = 0.048; *p*-for-interaction = 0.947). Cardiac death was infrequent, with no event in the SA DCB-based group, and MI and stroke were similarly distributed between strategies. Detailed event counts and effect estimates for all endpoints are provided in Table 3 (Figure S3).

#### 3.2.2. Stable Angina Versus Acute Coronary Syndrome Within Each Treatment Strategy

To clarify the source of this effect modification, SA and ACS were compared directly within each treatment strategy (Table 3; Figure S4). Within the DCB-based group, patients with SA had a significantly lower risk of MACCE than those with ACS (HR, 0.47; 95% CI, 0.31–0.69; *p* < 0.001), whereas within the DES-only group MACCE risk was virtually identical (HR, 0.99; 95% CI, 0.85–1.16; *p* = 0.911); this difference between strategies was significant (*p*-for-interaction < 0.001). A concordant pattern was observed for NACE (DCB-based HR, 0.46 [95% CI, 0.31–0.67]; DES-only HR, 0.94 [95% CI, 0.82–1.07]; *p*-for-interaction < 0.001) and for TVR (*p*-for-interaction = 0.006). Thus, the SA-versus-ACS prognostic gradient was confined to DCB-treated patients, indicating that the stronger DCB-versus-DES association in SA was driven primarily by outcomes in the DCB-based group. For MI, SA was associated with lower risk than ACS within the DES-only group (HR, 0.57; 95% CI, 0.37–0.88; *p* = 0.012) but not within the DCB-based group (HR, 0.73; 95% CI, 0.31–1.70; *p* = 0.466), without a significant interaction (*p*-for-interaction = 0.599). The remaining endpoints showed no significant SA-versus-ACS difference within either treatment group.

### 3.3. Independent Predictors of MACCE

In multivariable Cox models with all candidate variables entered simultaneously (Table S9), a DCB-based versus DES-only strategy was independently associated with lower MACCE in both the SA stratum (adjusted HR [aHR], 0.33; 95% CI, 0.23–0.49; *p* < 0.001) and the ACS stratum (aHR, 0.78; 95% CI, 0.63–0.97; *p* = 0.024). In SA, lower LVEF, higher serum creatinine, and RCA lesions were independently associated with higher risk, and ACC/AHA type B2/C lesions paradoxically with lower adjusted risk (aHR, 0.75; 95% CI, 0.57–0.99; *p* = 0.040). In ACS, older age, male sex, hypertension, previous PCI, lower LVEF, lower hemoglobin, higher serum creatinine, and left main lesions were independently associated with higher risk, and type B2/C lesions were again paradoxically associated with lower adjusted risk (aHR, 0.79; 95% CI, 0.65–0.96; *p* = 0.019).

### 3.4. Subgroup Analyses

The treatment effect on 3-year MACCE was directionally consistent across eight predefined subgroups in both cohorts, with overlapping confidence intervals and non-significant interactions for most comparisons (Figure S2). Two exceptions were noted: in SA, the benefit was more pronounced in lesions without ACC/AHA type B2/C complexity (*p*-for-interaction = 0.003), and in ACS it was confined to patients with an estimated glomerular filtration rate ≥ 60 mL/min/1.73 m^2^ (*p*-for-interaction = 0.002). Given the number of subgroups and limited events, these interactions are hypothesis-generating.

### 3.5. Sensitivity Analyses

The primary MACCE findings were robust across multivariable Cox adjustment, IPTW, E-values, 30-day landmark, and competing-risk analyses (Tables S4 and S5). In SA, multivariable Cox and IPTW estimates were 0.32 (95% CI, 0.22–0.47) and 0.26 (95% CI, 0.17–0.41); corresponding ACS estimates were 0.75 (95% CI, 0.61–0.93) and 0.76 (95% CI, 0.59–0.98). The E-value [20] was 5.56 in SA (3.52 for the CI bound nearest the null) and 2.06 in ACS (1.42 for the bound). No significant treatment-by-period interaction was observed in the landmark analysis (SA, *p*-for-interaction = 0.523; ACS, *p*-for-interaction = 0.104), and cause-specific and Fine–Gray subdistribution hazards were nearly identical, indicating that competing mortality did not materially affect inference. In additional analyses (Table S10), the association was of similar direction whether the DCB-based group was restricted to DCB-only PCI (SA: HR, 0.30; 95% CI, 0.19–0.49; ACS: HR, 0.78; 95% CI, 0.60–1.01) or to hybrid DCB–DES PCI (SA: HR, 0.43; 95% CI, 0.23–0.82; ACS: HR, 0.71; 95% CI, 0.51–1.00). Within the ACS stratum it persisted after exclusion of STEMI (HR, 0.68; 95% CI, 0.53–0.86) but was absent among patients presenting with STEMI (HR, 0.97; 95% CI, 0.56–1.70). The lower rate of major bleeding was not attenuated by additional adjustment for DAPT duration (SA: HR, 0.29; 95% CI, 0.14–0.63; ACS: HR, 0.30; 95% CI, 0.18–0.51).

## 4. Discussion

This overlap-weighted analysis of 11,522 patients from two multicenter registries who underwent PCI yielded three principal findings: First, a DCB-based strategy was associated with a lower 3-year risk of MACCE than a DES-only strategy in both clinical presentations, but the relative reduction was substantially greater in SA than in ACS; concordant gradients were observed for TVR and NACE. Second, complementary within-strategy analyses localized this effect modification: the prognostic advantage of SA over ACS was confined to patients treated with a DCB-based strategy and was essentially absent within the DES-only group. Third, the incidence of major bleeding was lower with the DCB-based strategy in both presentations. 

Unlike BASKET-SMALL 2 [7], which was limited to small-vessel disease and found no interaction for the composite endpoint, our larger real-world cohort encompassing broader lesion complexity showed a significant treatment-by-presentation interaction, with a stronger association of DCB-based PCI with lower MACCE in SA. Our findings also differ from a recent nationwide Japanese registry of single de novo ACS culprit lesions [5], which reported comparable DCB and DES outcomes; in our analysis, DCB-based PCI was associated with lower MACCE, TVR, and NACE even in ACS. This divergence likely reflects design differences: the Japanese registry had 1-year follow-up and was restricted to a single culprit lesion, whereas we assessed broader lesion complexity over 3 years. Our DCB-based group also comprised only patients with adequate lesion preparation who did not require rescue stenting, unlike the intention-to-treat REC-CAGEFREE I trial [21], in which DCB treatment did not achieve non-inferiority to upfront stenting; our findings therefore apply to successful DCB-based PCI rather than an unrestricted strategy. Since lesions that respond favorably to preparation may also carry a better prognosis whatever device is finally used, part of the advantage observed in SA may reflect this conditioning on procedural success.

The within-strategy results suggest a biologically plausible mechanism. Effective DCB therapy requires sustained drug contact with a receptive vessel wall during a single inflation; unlike a DES, which releases its agent from a permanent scaffold over weeks, a DCB has only one opportunity to transfer its drug [2,22]. In SA, the more stable, less thrombotic milieu may favor optimal lesion preparation and uniform drug transfer, and this leave-nothing-behind approach may permit positive vessel remodeling. In ACS, intraluminal thrombus may impair uniform drug transfer, uncertainty in culprit-vessel sizing may complicate balloon selection, and consensus cautions against DCB use in the presence of overt thrombus [10]. These substrate-related constraints, together with ongoing plaque instability, may explain why the prognostic advantage of SA over ACS was confined to DCB-treated patients, whose outcomes depend heavily on the quality of lesion preparation and drug delivery. Consistent with this account, the association was absent among patients presenting with STEMI, in whom thrombus burden is greatest, whereas it persisted once STEMI was excluded from the ACS stratum (Table S10).

Lesions with greater angiographic complexity, defined as ACC/AHA type B2/C, were paradoxically associated with a lower adjusted risk of MACCE in both strata (Table S9). This counterintuitive association is unlikely to reflect a protective effect of lesion complexity itself, but may instead reflect procedural selection within a successful DCB-based strategy. Complex lesions generally require rigorous preparation, careful angiographic optimization, judicious device selection, and a low threshold for bailout stenting [10]. Accordingly, patients with complex lesions who ultimately undergo successful DCB-based PCI may represent a selected subset of patients with well-prepared vessels and favorable acute procedural results. In line with this interpretation, the subgroup analysis in the SA stratum (Figure S2) suggested that the relative benefit of DCB-based treatment was the greatest in lesions without ACC/AHA type B2/C complexity, wherein achieving an adequate luminal result was likely less constrained. In the ACS stratum (Figure S2), the benefit of a DCB-based strategy was limited to patients with preserved renal function and was not apparent in those with estimated glomerular filtration rate below 60 mL/min/1.73 m^2^ (*p*-for-interaction = 0.002). Reduced renal function is accompanied by accelerated and diffuse atherosclerosis, medial calcification, endothelial dysfunction, and impaired vascular healing, which may create less receptive vessel walls for balloon-based drug delivery, thereby diluting the potential advantages of the DCB-based approach [23]. The presentation-dependent gradient is also unlikely to be a mere consequence of selective stenting of complex lesions, since estimates of similar direction were obtained whether the DCB-based group was confined to DCB-only procedures or to hybrid DCB–DES procedures (Table S10).

Within the DES-only group, ACS carried a higher MI risk than SA, a difference absent in the DCB-based group. Given infrequent events and a non-significant interaction (*p*-for-interaction = 0.599, Table 3), this is exploratory and likely reflects the higher baseline infarction propensity of ACS rather than a device effect, consistent with prior DES registry data identifying ACS presentation as an independent predictor of subsequent MI [24].

A DCB-based strategy was also associated with a lower risk of major bleeding (BARC 3–5) than a DES-only strategy in both presentations, with nearly identical estimates in the two strata (overlap-weighted HR, 0.32 each; *p*-for-interaction = 0.943; Table 3). Since major bleeding is a component of NACE, this reduction contributed to the more favorable NACE profile of the DCB-based strategy. However, DAPT duration and P2Y12 inhibitor choice were not randomized and remained imbalanced after weighting, particularly because the DES-only registry was restricted to clopidogrel-treated patients. Therefore, the findings for bleeding and NACE should be interpreted with caution and should not be attributed solely to device strategy. Observed DAPT duration was in fact comparable between the groups, and additional adjustment for it left the bleeding estimates essentially unchanged (Table S10); the difference nevertheless coincides with differences in P2Y12 regimen and in the source registries themselves, and should therefore be read as exploratory rather than as evidence of a device effect. Beta-blockers were likewise prescribed to fewer than half of the patients with SA (42.0% in the DCB-based and 50.7% in the DES-only group), a pattern that reflects the Korean practice of reserving beta-blockade for impaired systolic function, previous infarction, or arrhythmia rather than prescribing it universally, and one that accords with randomized evidence showing no reduction in death, reinfarction, or heart failure after myocardial infarction when the ejection fraction is preserved [25]. Discharge medications were kept out of the propensity-score models because they are recorded after the device decision and may lie on the causal pathway, so their differences persisted after weighting.

Taken together, these findings support considering a DCB-based strategy across the PCI spectrum, most strongly in stable presentations where elective timing permits meticulous lesion preparation, and in carefully selected ACS patients without overt thrombus and with preserved renal function. Because the analysis is observational, these results should inform rather than dictate device selection, and adequately powered randomized trials stratified by presentation are needed.

This study had several limitations. First, its observational, registry-based, nonrandomized design made it susceptible to residual and unmeasured confounding factors. The E-value was high in the SA stratum (5.56) but modest in the ACS stratum (2.06), indicating greater vulnerability of the ACS findings to unmeasured confounding factors. The two strategies were moreover drawn from separate registries rather than from a single cohort, so differences between the participating centers, their enrollment windows, and their institutional routines are not removed by weighting on measured covariates. The calendar periods of the two cohorts are a particular concern: DCB-based procedures were performed predominantly in the later years of the study period and DES-only procedures predominantly in the earlier years, so that device strategy and treatment era are closely aligned in this cohort. Calendar year was not entered into the propensity-score models, and secular changes in intravascular imaging, lesion-preparation technique, adjunctive pharmacotherapy, and follow-up practice therefore cannot be disentangled from the device comparison itself. The DCB-based cohort, contributed by five centers, may accordingly not represent the same population as the nationwide DES network, and the acute coronary syndrome estimate in particular should be read with that in mind. This limitation applies to every estimate reported here. Second, entry into the DCB-based group required an acceptable acute result after lesion preparation, whereas the DES-only group faced no equivalent filter; these findings therefore describe successfully completed DCB-based PCI, should not be generalized to an all-comer intention-to-treat DCB strategy, and may in part reflect this conditioning on procedural success. Third, subgroup and interaction analyses were exploratory and subject to multiplicity and limited events in some strata, which constrained precision. Fourth, antiplatelet regimens were not harmonized across the data sources: the PTRG-DES consortium [9] enrolled clopidogrel-treated recipients while excluding ticagrelor and prasugrel users, and treatment duration varied by registry. Therefore, inferences about bleeding and its contribution to NACE should be made with caution. Discharge medications were likewise not modeled and remained imbalanced after weighting. Finally, the cohort was predominantly derived from the Asian population, and its generalizability to other populations and DCB devices remains unclear.

## 5. Conclusions

In this large overlap-weighted cohort spanning patients with SA and ACS, a DCB-based strategy was associated with a lower risk of 3-year MACCE than a DES-only strategy in both presentations, with a stronger relative association in patients with SA. Within-strategy analyses localized this presentation-dependent gradient to the DCB-treated cohort, supporting the hypothesis that the outcomes of balloon-based drug delivery are influenced by the underlying lesions and clinical substrates. These findings suggest that the advantages of a leave-nothing-behind strategy may be the greatest in cases with stable presentations and support further randomized evaluations of DCB-based PCI in selected patients across the contemporary PCI spectrum. Because the comparison was non-randomized and drew on two separate registries, these conclusions should be regarded as hypothesis-generating.

## Figures and Tables

**Table 1:** Baseline Characteristics of the Stable Angina Cohort: DCB-based versus DES-only Strategy.

Variable	Unweighted (*n* = 4490)				Overlap-Weighted			
	DCB-Based (*n* = 898)	DES-Only (*n* = 3592)	*p* Value	SMD	DCB-Based	DES-Only	*p* Value	SMD
**Demographics and medical history**								
Age (years)	64.3 ± 9.7	64.6 ± 10.3	0.380	0.032	64.5 ± 9.8	64.5 ± 10.2	0.995	0.000
Male sex	708 (78.8)	2456 (68.4)	<0.001	0.239	428 (76.0)	428 (76.1)	0.987	0.001
Hypertension	649 (72.3)	2279 (63.4)	<0.001	0.190	389 (69.1)	389 (69.1)	0.991	0.001
Diabetes mellitus	437 (48.7)	1316 (36.6)	<0.001	0.245	250 (44.4)	250 (44.5)	0.984	0.001
Current smoking	207 (23.1)	823 (22.9)	0.965	0.003	132 (23.5)	132 (23.5)	0.991	0.001
Previous MI	103 (11.5)	254 (7.1)	<0.001	0.152	53 (9.3)	53 (9.4)	0.989	0.001
Previous PCI	234 (26.1)	462 (12.9)	<0.001	0.338	118 (20.9)	118 (20.9)	0.999	0.000
Previous stroke	126 (14.0)	287 (8.0)	<0.001	0.194	65 (11.6)	65 (11.6)	0.998	0.000
**Laboratory parameters**								
LVEF (%)	59.3 ± 9.5	60.8 ± 10.1	<0.001	0.155	59.8 ± 9.0	59.8 ± 10.6	0.995	0.000
Hemoglobin (g/dL)	13.7 ± 4.1	13.5 ± 1.8	0.277	0.048	13.6 ± 2.2	13.6 ± 1.9	0.999	0.000
Serum creatinine (mg/dL)	1.4 ± 1.9	1.0 ± 1.0	<0.001	0.211	1.2 ± 1.5	1.2 ± 1.4	0.996	0.000
**Discharge medications**								
Aspirin	895 (99.7)	3470 (96.6)	<0.001	0.228	561 (99.7)	542 (96.4)	<0.001	0.242
Clopidogrel	889 (99.0)	3592 (100.0)	<0.001	0.142	558 (99.1)	563 (100.0)	0.027	0.132
Ticagrelor	59 (6.6)	0	<0.001	0.375	37 (6.6)	0	<0.001	0.376
Prasugrel	9 (1.0)	0	<0.001	0.142	5 (0.9)	0	0.024	0.134
DAPT duration (days)	637.0 ± 903.4	625.7 ± 651.0	0.725	0.014	605.1 ± 906.1	681.0 ± 687.7	0.035	0.094
Beta-blocker	377 (42.0)	1822 (50.7)	<0.001	0.175	224 (39.8)	291 (51.8)	<0.001	0.242
ACEI or ARB	449 (50.0)	2147 (59.8)	<0.001	0.197	273 (48.6)	344 (61.2)	<0.001	0.255
Statin	875 (97.5)	3187 (88.7)	<0.001	0.354	546 (97.1)	495 (88.0)	<0.001	0.360
**Treatment strategy**								
DCB-only treatment	675 (75.2)	—	—	—	427 (75.9)	—	—	—
**Target lesion**								
Left main	93 (10.4)	195 (5.4)	<0.001	0.184	49 (8.7)	49 (8.8)	0.984	0.001
LAD	527 (58.7)	2183 (60.8)	0.269	0.043	333 (59.2)	333 (59.2)	1.000	0.000
LCX	377 (42.0)	1010 (28.1)	<0.001	0.294	220 (39.1)	220 (39.0)	0.986	0.001
RCA	289 (32.2)	1288 (35.9)	0.043	0.078	196 (34.8)	196 (34.8)	0.995	0.000
Multivessel PCI	305 (34.0)	932 (25.9)	<0.001	0.176	182 (32.4)	208 (37.0)	0.109	0.096
**Disease extent**								
1-vessel disease	340 (37.9)	2320 (64.6)	<0.001	0.555	255 (45.4)	256 (45.4)	0.992	0.001
2-vessel disease	325 (36.2)	910 (25.3)	<0.001	0.237	177 (31.5)	224 (39.9)	0.003	0.176
3-vessel disease	233 (25.9)	362 (10.1)	<0.001	0.422	130 (23.1)	83 (14.7)	<0.001	0.216
**Lesion complexity**								
Multivessel disease	558 (62.1)	1272 (35.4)	<0.001	0.556	307 (54.6)	307 (54.6)	0.992	0.001
Bifurcation lesion	342 (38.1)	395 (11.0)	<0.001	0.663	161 (28.7)	161 (28.6)	0.990	0.001
Chronic total occlusion	116 (12.9)	294 (8.2)	<0.001	0.155	63 (11.3)	64 (11.3)	0.995	0.000
ACC/AHA B2/C lesion	602 (67.0)	1647 (45.9)	<0.001	0.437	358 (63.6)	358 (63.6)	0.986	0.001
**Vessel/device counts**								
Total number of devices used	1.8 ± 1.0	1.6 ± 0.8	<0.001	0.149	1.7 ± 1.0	1.8 ± 0.9	0.210	0.054
Total device length (mm)	44.6 ± 28.1	37.1 ± 23.1	<0.001	0.293	43.6 ± 27.9	41.5 ± 25.0	0.068	0.079
Mean device diameter (mm)	2.8 ± 0.4	3.0 ± 0.4	<0.001	0.531	2.8 ± 0.4	2.8 ± 0.4	0.992	0.000
**DCB device characteristics**								
Total number of DCB used	1.4 ± 0.8	—	—	—	1.4 ± 0.8	—	—	—
Total DCB length (mm)	35.6 ± 22.7	—	—	—	34.8 ± 22.3	—	—	—
Mean DCB diameter (mm)	2.7 ± 0.4	—	—	—	2.8 ± 0.4	—	—	—
Small DCB used (diameter ≤ 2.5 mm)	431 (48.0)	—	—	—	251 (44.6)	—	—	—
**DES device characteristics**								
Total number of DES used	0.3 ± 0.6	1.6 ± 0.8	<0.001	1.786	0.3 ± 0.6	1.8 ± 0.9	<0.001	1.897
Total DES length (mm)	9.0 ± 18.3	37.1 ± 23.1	<0.001	1.347	8.8 ± 18.4	41.5 ± 25.0	<0.001	1.490
Mean DES diameter (mm)	3.1 ± 0.4	3.0 ± 0.4	<0.001	0.295	3.18 ± 0.46	2.83 ± 0.35	<0.001	0.850
Small DES used (diameter ≤ 2.5 mm)	21 (9.5)	865 (24.1)	<0.001	0.398	10 (1.8)	206 (36.6)	<0.001	0.986
**Follow-up**								
Follow-up duration (days)	1123.7 ± 900.3	967.5 ± 920.8	<0.001	0.171	1138.0 ± 911.4	1091.5 ± 941.8	0.231	0.050

Values are mean ± SD or *n* (%). Unweighted columns show raw values with *p* values from Student’s *t*-test (continuous) or chi-square test (categorical). All SMD values are absolute. For the DCB-based group, the unweighted percentage for small DES use is expressed among the patients who actually received a drug-eluting stent, whereas the overlap-weighted percentage refers to the group as a whole; expressed over the entire unweighted DCB-based group, the proportion is 2.3%. A complete-case strategy was applied without imputation; patients were excluded if any of the 22 baseline characteristics required for propensity score modeling were missing. ACC, American College of Cardiology; ACEI, angiotensin-converting enzyme inhibitor; AHA, American Heart Association; ARB, angiotensin receptor blocker; DAPT, dual antiplatelet therapy; DCB, drug-coated balloon; DES, drug-eluting stent; LAD, left anterior descending artery; LCX, left circumflex artery; LVEF, left ventricular ejection fraction; MI, myocardial infarction; NSTEMI, non-ST-segment elevation myocardial infarction; PCI, percutaneous coronary intervention; RCA, right coronary artery; SMD, standardized mean difference; STEMI, ST-segment elevation myocardial infarction.

**Table 2:** Baseline Characteristics of the Acute Coronary Syndrome Cohort: DCB-based versus DES-only Strategy.

Variable	Unweighted (*n* = 7032)				Overlap-Weighted			
	DCB-Based (*n* = 1549)	DES-Only (*n* = 5483)	*p* Value	SMD	DCB-Based	DES-Only	*p* Value	SMD
**Demographics and medical history**								
Age (years)	63.6 ± 11.7	64.6 ± 11.5	0.002	0.092	64.0 ± 11.8	64.0 ± 11.2	0.990	0.000
Male sex	1160 (74.9)	3573 (65.2)	<0.001	0.213	688 (71.0)	687 (71.0)	0.993	0.000
Hypertension	1016 (65.6)	3205 (58.5)	<0.001	0.147	617 (63.7)	617 (63.7)	0.999	0.000
Diabetes mellitus	629 (40.6)	1766 (32.2)	<0.001	0.175	369 (38.1)	369 (38.1)	0.992	0.000
Current smoking	470 (30.3)	1789 (32.6)	0.095	0.049	299 (30.9)	299 (30.9)	0.988	0.001
Previous MI	210 (13.6)	395 (7.2)	<0.001	0.209	110 (11.3)	109 (11.3)	0.987	0.001
Previous PCI	424 (27.4)	725 (13.2)	<0.001	0.357	213 (22.0)	213 (22.0)	0.991	0.000
Previous stroke	151 (9.7)	385 (7.0)	<0.001	0.098	85 (8.7)	85 (8.8)	0.993	0.000
**Laboratory parameters**								
LVEF (%)	56.6 ± 10.9	57.5 ± 10.7	0.005	0.081	57.0 ± 10.6	57.0 ± 11.2	0.992	0.000
Hemoglobin (g/dL)	13.7 ± 2.0	13.6 ± 1.9	0.026	0.065	13.6 ± 2.0	13.6 ± 1.9	0.993	0.000
Serum creatinine (mg/dL)	1.3 ± 1.5	1.1 ± 1.1	<0.001	0.123	1.2 ± 1.3	1.2 ± 1.3	0.992	0.000
**Discharge medications**								
Aspirin	1545 (99.7)	5359 (97.7)	<0.001	0.180	966 (99.8)	943 (97.4)	<0.001	0.201
Clopidogrel	1467 (94.7)	5483 (100.0)	<0.001	0.334	915 (94.5)	968 (100.0)	<0.001	0.340
Ticagrelor	346 (22.3)	0	<0.001	0.758	229 (23.6)	0	<0.001	0.786
Prasugrel	59 (3.8)	0	<0.001	0.281	33 (3.4)	0	<0.001	0.266
DAPT duration (days)	753.6 ± 961.7	739.2 ± 715.6	0.586	0.017	697.1 ± 915.8	792.5 ± 742.3	<0.001	0.114
Beta-blocker	912 (58.9)	3526 (64.3)	<0.001	0.112	568 (58.6)	592 (61.1)	0.262	0.051
ACEI or ARB	783 (50.5)	3372 (61.5)	<0.001	0.222	486 (50.3)	564 (58.2)	<0.001	0.160
Statin	1506 (97.2)	4791 (87.4)	<0.001	0.376	942 (97.3)	821 (84.8)	<0.001	0.450
**Clinical presentation**								
Unstable angina	1011 (65.3)	2624 (47.9)	<0.001	0.357	588 (60.8)	588 (60.8)	0.993	0.000
NSTEMI	335 (21.6)	1608 (29.3)	<0.001	0.177	235 (24.2)	235 (24.2)	0.999	0.000
STEMI	203 (13.1)	1251 (22.8)	<0.001	0.255	145 (15.0)	145 (15.0)	0.989	0.001
**Treatment strategy**								
DCB-only treatment	1006 (64.9)	—	—	—	641 (66.3)	—	—	—
**Target lesion**								
Left main	213 (13.8)	254 (4.6)	<0.001	0.320	94 (9.7)	94 (9.7)	0.992	0.000
LAD	946 (61.1)	3262 (59.5)	0.276	0.032	586 (60.6)	587 (60.6)	0.997	0.000
LCX	698 (45.1)	1694 (30.9)	<0.001	0.295	402 (41.5)	402 (41.5)	1.000	0.000
RCA	557 (36.0)	2212 (40.3)	0.002	0.090	370 (38.2)	370 (38.2)	0.998	0.000
Multivessel PCI	667 (43.1)	1616 (29.5)	<0.001	0.285	382 (39.5)	401 (41.4)	0.397	0.038
**Disease extent**								
1-vessel disease	492 (31.8)	3136 (57.2)	<0.001	0.529	370 (38.2)	369 (38.2)	0.991	0.001
2-vessel disease	575 (37.1)	1521 (27.7)	<0.001	0.201	329 (34.0)	398 (41.1)	0.001	0.146
3-vessel disease	482 (31.1)	826 (15.1)	<0.001	0.388	269 (27.8)	201 (20.8)	<0.001	0.164
**Lesion complexity**								
Multivessel disease	1057 (68.2)	2347 (42.8)	<0.001	0.533	598 (61.8)	598 (61.8)	0.991	0.001
Bifurcation lesion	617 (39.8)	695 (12.7)	<0.001	0.649	280 (28.9)	280 (28.9)	0.998	0.000
Chronic total occlusion	158 (10.2)	370 (6.7)	<0.001	0.124	90 (9.3)	90 (9.3)	0.993	0.000
ACC/AHA B2/C lesion	1168 (75.4)	3147 (57.4)	<0.001	0.388	692 (71.5)	692 (71.5)	0.988	0.001
**Vessel/device counts**								
Total number of devices used	1.9 ± 1.0	1.6 ± 0.8	<0.001	0.263	1.8 ± 1.0	1.8 ± 0.8	0.097	0.055
Total device length (mm)	47.5 ± 29.8	36.1 ± 22.3	<0.001	0.431	46.2 ± 29.2	39.5 ± 24.0	<0.001	0.250
Mean device diameter (mm)	2.8 ± 0.4	3.0 ± 0.5	<0.001	0.427	2.9 ± 0.4	2.9 ± 0.4	0.997	0.000
**DCB device characteristics**								
Total number of DCB used	1.4 ± 0.7	—	—	—	1.3 ± 0.7	—	—	—
Total DCB length (mm)	34.4 ± 21.6	—	—	—	33.6 ± 20.9	—	—	—
Mean DCB diameter (mm)	2.7 ± 0.4	—	—	—	2.8 ± 0.4	—	—	—
Small DCB used (diameter ≤ 2.5 mm)	758 (48.9)	—	—	—	443 (45.8)	—	—	—
**DES device characteristics**								
Total number of DES used	0.5 ± 0.8	1.6 ± 0.8	<0.001	1.432	0.5 ± 0.8	1.8 ± 0.8	<0.001	1.586
Total DES length (mm)	13.1 ± 22.8	36.1 ± 22.3	<0.001	1.019	12.5 ± 22.3	39.5 ± 24.0	<0.001	1.164
Mean DES diameter (mm)	3.2 ± 0.5	3.0 ± 0.5	<0.001	0.318	3.18 ± 0.48	2.87 ± 0.39	<0.001	0.727
Small DES used (diameter ≤ 2.5 mm)	49 (9.1)	1305 (23.8)	<0.001	0.404	28 (2.9)	334 (34.6)	<0.001	0.890
**Follow-up**								
Follow-up duration (days)	1305.3 ± 962.6	1171.9 ± 992.5	<0.001	0.136	1287.7 ± 957.2	1300.5 ± 1004.8	0.682	0.013

Values are mean ± SD or *n* (%). Unweighted columns show raw values with *p* values from Student’s *t*-test (continuous) or chi-square test (categorical). All SMD values are absolute. For the DCB-based group, the unweighted percentage for small DES use is expressed among the patients who actually received a drug-eluting stent, whereas the overlap-weighted percentage refers to the group as a whole; expressed over the entire unweighted DCB-based group, the proportion is 3.2%. A complete-case strategy was applied without imputation; patients were excluded if any of the 22 baseline characteristics required for propensity score modeling were missing. ACC, American College of Cardiology; ACEI, angiotensin-converting enzyme inhibitor; AHA, American Heart Association; ARB, angiotensin receptor blocker; DAPT, dual antiplatelet therapy; DCB, drug-coated balloon; DES, drug-eluting stent; LAD, left anterior descending artery; LCX, left circumflex artery; LVEF, left ventricular ejection fraction; MI, myocardial infarction; NSTEMI, non-ST-segment elevation myocardial infarction; PCI, percutaneous coronary intervention; RCA, right coronary artery; SMD, standardized mean difference; STEMI, ST-segment elevation myocardial infarction.

**Table 3:** Three-Year Clinical Outcomes.

(**A**) By treatment strategy within each clinical stratum (DCB-based versus DES-only)											
	SA (*n* = 4490)					ACS (*n* = 7032)					
Endpoint	DCB (*n* = 898)	DES (*n* = 3592)	Log-rank *p*	Unadjusted HR (95% CI); *p*	OW HR * (95% CI); *p*	DCB (*n* = 1549)	DES (*n* = 5483)	Log-rank *p*	Unadjusted HR (95% CI); *p*	OW HR * (95% CI); *p*	*p*-for-interaction
MACCE	33 (3.7)	282 (7.9)	<0.001	0.37 (0.26–0.53); <0.001	0.33 (0.22–0.49); <0.001	134 (8.7)	463 (8.4)	0.201	0.88 (0.73–1.07); 0.202	0.73 (0.59–0.91); 0.006	<0.001
Cardiac death	0	40 (1.1)	<0.001	—	—	34 (2.2)	82 (1.5)	0.192	1.31 (0.87–1.95); 0.193	1.05 (0.67–1.67); 0.822	—
Myocardial infarction	8 (0.9)	28 (0.8)	0.750	0.88 (0.40–1.94); 0.751	0.84 (0.34–2.10); 0.715	19 (1.2)	87 (1.6)	0.096	0.66 (0.40–1.08); 0.098	0.67 (0.39–1.15); 0.142	0.616
TVR	21 (2.3)	195 (5.4)	<0.001	0.34 (0.22–0.53); <0.001	0.34 (0.21–0.56); <0.001	79 (5.1)	301 (5.5)	0.066	0.79 (0.62–1.02); 0.066	0.64 (0.48–0.85); 0.002	0.037
TL or ST	1 (0.1)	15 (0.4)	0.168	0.27 (0.04–2.01); 0.200	0.28 (0.04–2.21); 0.227	3 (0.2)	32 (0.6)	0.052	0.33 (0.10–1.07); 0.065	0.30 (0.09–0.99); 0.048	0.947
Stroke	10 (1.1)	48 (1.3)	0.260	0.68 (0.34–1.34); 0.263	0.51 (0.23–1.09); 0.081	22 (1.4)	66 (1.2)	0.877	1.04 (0.64–1.69); 0.877	0.82 (0.47–1.42); 0.470	0.348
Major bleeding (BARC 3–5)	8 (0.9)	89 (2.5)	<0.001	0.32 (0.15–0.65); 0.002	0.32 (0.15–0.69); 0.003	19 (1.2)	180 (3.3)	<0.001	0.34 (0.21–0.55); <0.001	0.32 (0.19–0.53); <0.001	0.943
NACE	36 (4.0)	347 (9.7)	<0.001	0.33 (0.24–0.47); <0.001	0.30 (0.20–0.43); <0.001	150 (9.7)	597 (10.9)	0.004	0.77 (0.64–0.92); 0.004	0.66 (0.54–0.81); <0.001	<0.001
(**B**) By clinical stratum within each treatment group (SA versus ACS)											
	DCB-based (*n* = 2447)					DES-only (*n* = 9075)					
Endpoint	SA (*n* = 898)	ACS (*n* = 1549)	Log-rank *p*	Unadjusted HR (95% CI); *p*	OW HR * (95% CI); *p*	SA (*n* = 3592)	ACS (*n* = 5483)	Log-rank *p*	Unadjusted HR (95% CI); *p*	OW HR * (95% CI); *p*	*p*-for-interaction
MACCE	33 (3.7)	134 (8.7)	<0.001	0.44 (0.30–0.64); <0.001	0.47 (0.31–0.69); <0.001	282 (7.9)	463 (8.4)	0.883	0.99 (0.85–1.15); 0.884	0.99 (0.85–1.16); 0.911	<0.001
Cardiac death	0	34 (2.2)	<0.001	—	—	40 (1.1)	82 (1.5)	0.220	0.79 (0.54–1.15); 0.221	1.02 (0.69–1.52); 0.910	—
Myocardial infarction	8 (0.9)	19 (1.2)	0.501	0.75 (0.33–1.72); 0.502	0.73 (0.31–1.70); 0.466	28 (0.8)	87 (1.6)	0.003	0.53 (0.35–0.81); 0.004	0.57 (0.37–0.88); 0.012	0.599
TVR	21 (2.3)	79 (5.1)	0.002	0.47 (0.29–0.76); 0.002	0.48 (0.29–0.78); 0.003	195 (5.4)	301 (5.5)	0.588	1.05 (0.88–1.26); 0.588	0.98 (0.82–1.18); 0.841	0.006
TL or ST	1 (0.1)	3 (0.2)	0.647	0.59 (0.06–5.70); 0.650	0.69 (0.07–6.41); 0.745	15 (0.4)	32 (0.6)	0.279	0.71 (0.39–1.32); 0.281	0.85 (0.45–1.60); 0.613	0.846
Stroke	10 (1.1)	22 (1.4)	0.689	0.86 (0.41–1.81); 0.689	0.81 (0.37–1.77); 0.595	48 (1.3)	66 (1.2)	0.361	1.19 (0.82–1.73); 0.361	1.27 (0.86–1.88); 0.220	0.252
Major bleeding (BARC 3–5)	8 (0.9)	19 (1.2)	0.510	0.76 (0.33–1.73); 0.511	0.88 (0.37–2.08); 0.771	89 (2.5)	180 (3.3)	0.056	0.78 (0.61–1.01); 0.057	0.81 (0.62–1.05); 0.109	0.880
NACE	36 (4.0)	150 (9.7)	<0.001	0.42 (0.29–0.61); <0.001	0.46 (0.31–0.67); <0.001	347 (9.7)	597 (10.9)	0.298	0.93 (0.82–1.06); 0.298	0.94 (0.82–1.07); 0.335	<0.001

* Overlap-weighted (OW) hazard ratios were estimated using overlap weights derived from propensity score models comprising 20 covariates for the stable angina stratum (age, male sex, hypertension, diabetes mellitus, current smoking, previous MI, previous PCI, previous stroke, LVEF, hemoglobin, serum creatinine, left main disease, LAD, LCX, RCA, multivessel disease, bifurcation lesion, chronic total occlusion, ACC/AHA B2/C lesion, and mean device diameter) and 22 covariates for the acute coronary syndrome stratum (the above 20 plus NSTEMI and STEMI; unstable angina = reference). Robust (sandwich) standard errors were used to account for weighting. (**A**) Overlap weights were derived within each clinical stratum. Hazard ratios are reported with the DCB-based group as the comparison group and the DES-only group as the reference; values < 1 therefore indicate a lower event rate with a DCB-based than with a DES-only strategy. The *p* value for interaction was estimated by adding a treatment-by-stratum interaction term to the OW Cox model fitted on the pooled cohort using the within-stratum overlap weights. (**B**) Overlap weights were re-estimated within each treatment group. Hazard ratios are reported with the stable angina stratum as the comparison group and the acute coronary syndrome stratum as the reference; values < 1 therefore indicate a lower event rate in stable angina than in acute coronary syndrome. The *p* value for interaction is identical to that reported in (**A**) (treatment-by-stratum interaction term in the pooled OW Cox model) and is reproduced for ease of reference. ACC/AHA, American College of Cardiology/American Heart Association; ACS, acute coronary syndrome; CI, confidence interval; DCB, drug-coated balloon; DES, drug-eluting stent; HR, hazard ratio; LAD, left anterior descending coronary artery; LCX, left circumflex artery; LVEF, left ventricular ejection fraction; MACCE, major adverse cardiac and cerebrovascular events; MI, myocardial infarction; NACE, net adverse clinical events; NSTEMI, non-ST-segment elevation myocardial infarction; OW, overlap weighting; PCI, percutaneous coronary intervention; RCA, right coronary artery; SA, stable angina; ST, stent thrombosis; STEMI, ST-segment elevation myocardial infarction; TL, target lesion; TVR, target-vessel revascularization.

## Data Availability

The data supporting the findings of this study are derived from the REAL-DCB registry (NCT04619277) and an investigator-initiated multicenter cohort for the drug-coated balloon-based group, and from the PTRG-DES Consortium (NCT04734028) for the drug-eluting stent-only group. Owing to the nature of these registries and institutional privacy regulations, the raw data are not publicly available. However, de-identified data may be made available from the corresponding author upon reasonable request and with the permission of the respective registry steering committees.

## Supplementary Materials

The following supporting information can be downloaded at: https://www.mdpi.com/article/10.3390/jcm15166140/s1. Supplementary Methods; Table S1: Multicollinearity diagnostics for the candidate covariates considered for the propensity score models—overall cohort; Table S2: Multicollinearity diagnostics for the 20 covariates included in the propensity score model—stable angina cohort; Table S3: Multicollinearity diagnostics for the 22 covariates included in the propensity score model—acute coronary syndrome cohort; Table S4: Sensitivity analyses for the effect of DCB-based versus DES-only PCI on 3-year MACCE in the stable angina cohort; Table S5: Sensitivity analyses for the effect of DCB-based versus DES-only PCI on 3-year MACCE in the acute coronary syndrome cohort; Table S6: Baseline characteristics of the overall cohort: DCB-based versus DES-only strategy; Table S7: Baseline characteristics within the DCB-based group: stable angina versus acute coronary syndrome; Table S8: Baseline characteristics within the DES-only group: stable angina versus acute coronary syndrome; Table S9: Unadjusted and adjusted predictors of 3-year MACCE by clinical presentation; Table S10: Additional sensitivity analyses of 3-year outcomes: composition of the DCB-based strategy, influence of STEMI, and bleeding in relation to DAPT duration; Figure S1: Propensity score distribution and covariate balance in the stable angina cohort and in the acute coronary syndrome cohort; Figure S2: Subgroup analysis of DCB-based versus DES-only PCI for 3-year MACCE (overlap-weighted); Figure S3: Three-year cumulative incidence of clinical outcomes that did not differ significantly between treatment strategies, stratified by clinical presentation; Figure S4: Three-year cumulative incidence of clinical outcomes comparing stable angina with acute coronary syndrome within each treatment group.
