# Supplementary material for: Drug-Coated Balloon-Based Versus Drug-Eluting Stent-Only Strategy in Stable Angina and Acute Coronary Syndrome: A Propensity-Score Overlap-Weighted Analysis
Source: J Clin Med. 2026 Aug 7;15(16):6140. doi: 10.3390/jcm15166140 (PMC13513134; doi:10.3390/jcm15166140)
Supplement: Supplementary file 1 [file jcm-15-06140-s001.zip › jcm-4445308-supplementary.pdf]

Supplementary Online Contents

Supplementary methods

Table S1 Multicollinearity Diagnostics for the Candidate Covariates Considered for the Propensity Score Models — Overall Cohort

Table S2 Multicollinearity Diagnostics for the 20 Covariates Included in the Propensity Score Model — Stable Angina Cohort

Table S3 Multicollinearity Diagnostics for the 22 Covariates Included in the Propensity Score Model — Acute Coronary Syndrome Cohort

Table S4 Sensitivity Analyses for the Effect of DCB-based versus DES-only PCI on 3-Year MACCE in the Stable Angina Cohort

Table S5 Sensitivity Analyses for the Effect of DCB-based versus DES-only PCI on 3-Year MACCE in the Acute Coronary Syndrome Cohort

Table S6 Baseline Characteristics of the Overall Cohort: DCB-based versus DES-only Strategy

Table S7 Baseline Characteristics within the DCB-based Group: Stable Angina versus Acute Coronary Syndrome

Table S8 Baseline Characteristics within the DES-only Group: Stable Angina versus Acute Coronary Syndrome

Table S9 Unadjusted and Adjusted Predictors of 3-Year MACCE by Clinical Presentation

Table S10 Additional Sensitivity Analyses of 3-Year Outcomes: Composition of the DCB-based Strategy, Influence of STEMI, and Bleeding in Relation to DAPT Duration

Figure S1 Propensity score distribution and covariate balance in the Stable Angina cohort (A) and in the Acute Coronary Syndrome cohort (B)

Figure S2 Subgroup analysis of DCB-based versus DES-only PCI for 3-year MACCE (overlap-weighted)

Figure S3 Three-year cumulative incidence of clinical outcomes that did not differ significantly between a drug-coated balloon (DCB)–based strategy and a drug-eluting stent

Figure S4 Three-year cumulative incidence of clinical outcomes comparing stable angina (SA; green) with acute coronary syndrome (ACS; orange) within each treatment group

Supplementary methods

Lesion preparation and device details

In the DCB-based strategy, the procedural sequence followed contemporary consensus statements for coronary DCB use [10,11]. Before DCB treatment, lesion preparation was carried out with appropriately sized semi-compliant, non-compliant, or specialty balloons, targeting a balloon-to-artery ratio of at least 0.8 to 1.0. Adequate lesion preparation was required before DCB inflation and was defined as residual diameter stenosis no greater than 30%, Thrombolysis in Myocardial Infarction (TIMI) grade 3 antegrade flow, and no flow-limiting dissection. Once these criteria were met, a paclitaxel–iopromide-coated balloon (3.0 $\mu g/mm^2$; SeQuent Please, B. Braun, Melsungen, Germany) was inflated at nominal pressure for ≥ 60 seconds, with sizing chosen to extend at least 2 mm beyond both ends of the pre-dilated segment to reduce geographic miss. In cases of inadequate lesion preparation, including residual stenosis > 30%, flow-limiting dissection, or hemodynamic instability, the lesion was treated with DES implantation without subsequent DCB application [8,11]. In a hybrid PCI, a DCB was used at one lesion or segment and a DES at another. Patients with unsuccessful lesion preparation or those requiring bailout stenting within the intended DCB-treated lesion were excluded. Final angiography was performed at least 5 minutes after intracoronary vasodilator injection to rule out late vessel closure.

Covariate selection and propensity-score model

Candidate covariates were selected according to established propensity-score methodology, prioritizing variables with pathophysiological or prognostic relevance after PCI and potential influence on treatment assignment, while emphasizing outcome-associated covariates rather than variables associated only with treatment selection [17-19]. The propensity-score model in the SA stratum comprised 20 covariates: age, male sex, hypertension, diabetes mellitus,

current smoking, previous MI, previous PCI, previous stroke, left ventricular ejection fraction, hemoglobin level, serum creatinine level, left main lesion, left anterior descending artery lesion, left circumflex artery lesion, right coronary artery lesion, multivessel disease, bifurcation lesion, chronic total occlusion, American College of Cardiology/American Heart Association type B2/C lesion, and mean device diameter. In the ACS stratum, the same covariates were supplemented with two indicator terms distinguishing NSTEMI and STEMI from unstable angina (the reference category), yielding 22 covariates. The total number of devices and total device length were excluded because their variance inflation factors exceeded 5; mean device diameter, which was independent of both (variance inflation factor approximately 1.2), was retained. The complete covariate list and collinearity diagnostics are provided in Tables S1 to S3.

Sensitivity and subgroup analyses

Robustness was examined in five predefined sensitivity analyses: (1) a conventional multivariable Cox proportional-hazard model adjusted for all propensity-score covariates; (2) inverse probability of treatment weighting (IPTW) with stabilized weights; (3) computation of the E-value to quantify the minimum strength of association an unmeasured confounder would need with both treatment and outcome to explain the observed effect [20]; (4) a 30-day landmark analysis partitioning follow-up into early (0–30 days) and late (30 days–3 years) periods, with a treatment-by-period interaction term; and (5) examination of the individual nonfatal composite components using both cause-specific and Fine–Gray subdistribution hazard models, with cardiac death as the competing event. Predefined subgroup analyses were conducted across eight clinically relevant strata (age, sex, diabetes mellitus, LVEF, estimated glomerular filtration rate, multivessel disease, left main involvement, and ACC/AHA lesion complexity), with multiplicative interaction tested on the log-hazard scale.

Table S1 Multicollinearity Diagnostics for the Candidate Covariates Considered for the Propensity Score Models — Overall Cohort

	Variance Inflation Factors	Tolerance	Condition Index
Age	1.449	0.690	1.000
Male sex	1.373	0.728	2.820
Hypertension	1.143	0.875	3.190
Diabetes mellitus	1.097	0.912	3.420
Current smoking	1.289	0.776	3.490
Previous MI	1.513	0.661	3.910
Previous PCI	1.537	0.651	3.960
Previous stroke	1.049	0.953	4.070
LVEF	1.079	0.926	4.210
Hemoglobin	1.551	0.645	4.410
Serum creatinine	1.186	0.843	4.860
Left main lesion	1.154	0.867	4.980
LAD lesion	2.058	0.486	5.480
LCX lesion	1.996	0.501	5.550
RCA lesion	2.129	0.470	6.370
Multivessel disease	1.804	0.554	7.380
Bifurcation lesion	1.171	0.854	7.990
Chronic total occlusion	1.036	0.965	9.970
ACC/AHA type B2/C	1.335	0.749	12.620
Total number of devices used	1.163	0.860	18.050
Total device length	1.702	0.588	21.010
Mean device diameter	1.078	0.928	26.880

ACC, American College of Cardiology; AHA, American Heart Association; LAD, left anterior descending artery; LCX, left circumflex artery; LVEF, left ventricular ejection fraction; MI, myocardial infarction; PCI, percutaneous coronary intervention; RCA, right coronary artery.

Table S2 Multicollinearity Diagnostics for the 20 Covariates Included in the Propensity Score Model — Stable Angina Cohort

	Variance Inflation Factors	Tolerance	Condition Index
Age	1.176	0.851	1.000
Male sex	1.233	0.811	2.892
Hypertension	1.094	0.914	2.971
Diabetes mellitus	1.074	0.931	3.309
Current smoking	1.125	0.889	3.400
Previous MI	1.305	0.766	3.531
Previous PCI	1.270	0.787	3.696
Previous stroke	1.019	0.981	3.793
LVEF	1.101	0.908	3.996
Hemoglobin	1.204	0.831	4.197
Serum creatinine	1.101	0.909	4.552
Left main lesion	1.236	0.809	4.641
LAD lesion	1.941	0.515	4.768
LCX lesion	1.964	0.509	5.812
RCA lesion	1.953	0.512	6.762
Multivessel disease	2.212	0.452	7.062
Bifurcation lesion	1.185	0.844	10.435
Chronic total occlusion	1.054	0.949	19.339
ACC/AHA type B2/C	1.580	0.633	21.555
Total number of devices used	6.830	0.146	23.190
Total device length	6.656	0.150	25.015
Mean device diameter	1.152	0.868	23.167

Total number of devices used and total device length had variance inflation factors exceeding 5 and were therefore excluded from the propensity score model, leaving 20 covariates. ACC, American College of Cardiology; AHA, American Heart Association; LAD, left anterior descending artery; LCX, left circumflex artery; LVEF, left ventricular ejection fraction; MI, myocardial infarction; PCI, percutaneous coronary intervention; RCA, right coronary artery.

Table S3 Multicollinearity Diagnostics for the 22 Covariates Included in the Propensity Score Model — Acute Coronary Syndrome Cohort

	Variance Inflation Factors	Tolerance	Condition Index
Age	1.363	0.734	1.000
Male sex	1.285	0.778	2.858
Hypertension	1.124	0.889	3.074
Diabetes mellitus	1.085	0.922	3.254
Current smoking	1.251	0.799	3.492
Previous MI	1.406	0.711	3.583
Previous PCI	1.443	0.693	3.835
Previous stroke	1.036	0.965	3.844
LVEF	1.226	0.816	4.026
Hemoglobin	1.504	0.665	4.256
Serum creatinine	1.163	0.860	4.447
NSTEMI	1.237	0.808	4.726
STEMI	1.384	0.722	4.893
Left main lesion	1.169	0.855	5.147
LAD lesion	1.680	0.595	5.433
LCX lesion	1.807	0.554	5.990
RCA lesion	1.729	0.578	6.953
Multivessel disease	2.004	0.499	7.260
Bifurcation lesion	1.194	0.838	10.104
Chronic total occlusion	1.042	0.960	19.457
ACC/AHA type B2/C	1.435	0.697	22.551
Total number of devices used	6.232	0.160	23.857
Total device length	5.606	0.178	24.763
Mean device diameter	1.164	0.859	27.006

Total number of devices used and total device length had variance inflation factors exceeding 5 and were therefore excluded from the propensity score model, leaving 22 covariates. ACC, American College of Cardiology; AHA, American Heart Association; LAD, left anterior descending artery; LCX, left circumflex artery; LVEF, left ventricular ejection fraction; MI, myocardial infarction; NSTEMI, non–ST-segment elevation myocardial infarction; PCI, percutaneous coronary intervention; RCA, right coronary artery; STEMI, ST-segment elevation myocardial infarction.

Table S4 Sensitivity Analyses for the Effect of DCB-based versus DES-only PCI on 3-Year MACCE in the Stable Angina Cohort

A. Treatment-effect estimators

Analysis	HR (95% CI)	*p* value
Overlap-weighted Cox (primary, Table 3)	0.33 (0.22–0.49)	<0.001
Multivariable Cox	0.32 (0.22–0.47)	<0.001
Inverse probability of treatment weighting (IPTW) Cox	0.26 (0.17–0.41)	<0.001

B. E-value (unmeasured confounding)

The E-value quantifies the minimum strength of association (on the risk-ratio scale) that an unmeasured confounder, over and above the measured covariates, would need to have with both the exposure (DCB-based PCI) and the outcome (MACCE) to fully explain away the observed association. For the primary OW Cox estimate, the E-value is 5.56 for the point estimate and 3.52 for the confidence-interval bound closest to the null.

C. Landmark analysis (30-day landmark)

Period	OW HR (95% CI)	*p*	*p*-for-interaction
Early (0–30 days)	0.52 (0.12–2.24)	0.382	0.523
Late (30 days – 3 years)	0.32 (0.21–0.48)	<0.001	

Landmark analysis split follow-up at 30 days. The early period assessed periprocedural events; the late period (conditional on event-free survival past day 30) assessed the persistence of the DCB-associated lower risk. The interaction P value tests whether the DCB association differed between periods on the log-hazard scale.

D. Competing risk analysis (cardiac death as competing event)

Outcome	Events / Competing	Cause-specific HR (95% CI); *p*	Fine-Gray sHR (95% CI); *p*
Myocardial infarction	36 / 36	0.84 (0.34–2.10); $p = 0.715$	0.84 (0.34–2.10); $p = 0.715$
Stroke	58 / 38	0.51 (0.23–1.09); $p = 0.081$	0.51 (0.23–1.09); $p = 0.081$
TL or stent thrombosis	16 / 36	0.28 (0.04–2.21); $p = 0.227$	0.28 (0.04–2.21); $p = 0.227$
Target-vessel revascularization	216 / 37	0.34 (0.21–0.56); $p < 0.001$	0.34 (0.21–0.56); $p < 0.001$

Cause-specific Cox treats cardiac death as censoring; Fine-Gray subdistribution hazard treats cardiac death as a competing event that keeps the subject in the risk set. Concordance between cause-specific and subdistribution estimates indicates that competing risk does not materially affect inference.

All analyses pertain to the Stable Angina stratum (n = 4490). Outcomes censored at 3 years (1095 days). OW analysis used overlap weighting on covariates from the propensity score model (see Table 2 footnote). IPTW analysis used inverse probability of treatment weighting with stabilized weights for average treatment effect estimation. The multivariable Cox model included all propensity-score covariates as adjustment variables. CI, confidence interval; DCB, drug-coated balloon; DES, drug-eluting stent; HR, hazard ratio; IPTW, inverse probability of treatment weighting; MACCE, major adverse cardiac and cerebrovascular events; OW, overlap weighting; PCI, percutaneous coronary intervention; sHR, subdistribution hazard ratio; TL, target lesion.

Table S5 Sensitivity Analyses for the Effect of DCB-based versus DES-only PCI on 3-Year MACCE in the Acute Coronary Syndrome Cohort

A. Treatment-effect estimators

Analysis	HR (95% CI)	*p* value
Overlap-weighted Cox (primary, Table 3)	0.73 (0.59–0.91)	0.006
Multivariable Cox	0.75 (0.61–0.93)	0.009
Inverse probability of treatment weighting (IPTW) Cox	0.76 (0.59–0.98)	0.038

B. E-value (unmeasured confounding)

The E-value quantifies the minimum strength of association (on the risk-ratio scale) that an unmeasured confounder, over and above the measured covariates, would need to have with both the exposure (DCB-based PCI) and the outcome (MACCE) to fully explain away the observed association. For the primary OW Cox estimate, the E-value is 2.06 for the point estimate and 1.42 for the confidence-interval bound closest to the null.

C. Landmark analysis (30-day landmark)

Period	OW HR (95% CI)	*p*	*p*-for-interaction
Early (0–30 days)	1.20 (0.65–2.19)	0.561	0.104
Late (30 days – 3 years)	0.69 (0.54–0.87)	0.002	

Landmark analysis split follow-up at 30 days. The early period assessed periprocedural events; the late period (conditional on event-free survival past day 30) assessed the persistence of the DCB-associated lower risk. The interaction P value tests whether the DCB association differed between periods on the log-hazard scale.

D. Competing risk analysis (cardiac death as competing event)

Outcome	Events / Competing	Cause-specific HR (95% CI); *p*	Fine-Gray sHR (95% CI); *p*
Myocardial infarction	106 / 105	0.67 (0.39–1.15); *p* = 0.142	0.67 (0.39–1.15); *p* = 0.142
Stroke	88 / 111	0.82 (0.47–1.42); *p* = 0.472	0.82 (0.47–1.42); *p* = 0.472
TL or stent thrombosis	35 / 105	0.30 (0.09–0.99); *p* = 0.048	0.30 (0.09–0.99); *p* = 0.048
Target-vessel revascularization	380 / 105	0.64 (0.48–0.85); *p* = 0.002	0.64 (0.48–0.85); *p* = 0.002

Cause-specific Cox treats cardiac death as censoring; Fine-Gray subdistribution hazard treats cardiac death as a competing event that keeps the subject in the risk set. Concordance between cause-specific and subdistribution estimates indicates that competing risk does not materially affect inference.

All analyses pertain to the Acute Coronary Syndrome stratum (n = 7032). Outcomes censored at 3 years (1095 days). OW analysis used overlap weighting on covariates from the propensity score model (see Table 3 footnote). IPTW analysis used inverse probability of treatment weighting with stabilized weights for average treatment effect estimation. The multivariable Cox model included all propensity-score covariates as adjustment variables. CI, confidence interval; DCB, drug-coated balloon; DES, drug-eluting stent; HR, hazard ratio; IPTW, inverse probability of treatment weighting; MACCE, major adverse cardiac and cerebrovascular events; OW, overlap weighting; PCI, percutaneous coronary intervention; sHR, subdistribution hazard ratio; TL, target lesion.

Table S6 Baseline Characteristics of the Overall Cohort: DCB-based versus DES-only Strategy

Variable	Unweighted (n = 11,522)				Overlap-Weighted			
	DCB-based (n = 2447)	DES-only (n = 9075)	*p* value	SMD	DCB-based	DES-only	*p* value	SMD
**Demographics and medical history**								
Age (years)	63.8 ± 11.0	64.6 ± 11.1	0.002	0.072	64.1 ± 11.1	64.1 ± 10.8	0.990	0.000
Male sex	1868 (76.3)	6029 (66.4)	<0.001	0.220	1129 (73.0)	1129 (72.9)	1.000	0.001
Hypertension	1665 (68.0)	5484 (60.4)	<0.001	0.159	1016 (65.7)	1016 (65.7)	1.000	0.000
Diabetes mellitus	1066 (43.6)	3082 (34.0)	<0.001	0.198	626 (40.5)	626 (40.4)	1.000	0.001
Current smoking	677 (27.7)	2612 (28.8)	0.289	0.025	435 (28.1)	435 (28.1)	1.000	0.000
Previous MI	313 (12.8)	649 (7.2)	<0.001	0.189	165 (10.6)	165 (10.6)	1.000	0.000
Previous PCI	658 (26.9)	1187 (13.1)	<0.001	0.351	333 (21.5)	333 (21.5)	1.000	0.000
Previous stroke	277 (11.3)	672 (7.4)	<0.001	0.135	152 (9.8)	152 (9.8)	1.000	0.000
**Laboratory parameters**								
LVEF (%)	57.6 ± 10.5	58.8 ± 10.6	<0.001	0.114	58.1 ± 10.1	58.1 ± 11.1	0.993	0.000
Hemoglobin (g/dL)	13.6 ± 2.0	13.5 ± 1.9	0.039	0.048	13.6 ± 2.0	13.6 ± 1.9	0.995	0.000
Serum creatinine (mg/dL)	1.3 ± 1.6	1.1 ± 1.0	<0.001	0.159	1.2 ± 1.4	1.2 ± 1.4	0.998	0.000
**Discharge medications**								
Aspirin	2440 (99.7)	8829 (97.3)	<0.001	0.201	1544 (99.7)	1501 (97.0)	<0.001	0.220
Clopidogrel	2356 (96.3)	9075 (100.0)	<0.001	0.278	1488 (96.2)	1548 (100.0)	<0.001	0.282
Ticagrelor	405 (16.6)	0	<0.001	0.630	270 (17.4)	0	<0.001	0.649
Prasugrel	68 (2.8)	0	<0.001	0.239	40 (2.6)	0	<0.001	0.230
DAPT duration (days)	710.9 ± 942.1	694.3 ± 692.9	0.418	0.020	666.9 ± 917.7	750.3 ± 724.9	<0.001	0.101
Beta-blocker	1289 (52.7)	5348 (58.9)	<0.001	0.126	800 (51.7)	891 (57.6)	<0.001	0.118
ACEI or ARB	1232 (50.3)	5519 (60.8)	<0.001	0.212	768 (49.6)	922 (59.6)	<0.001	0.200

Statin	2381 (97.3)	7978 (87.9)	<0.001	0.367	1505 (97.3)	1332 (86.1)	<0.001	0.417
**Clinical presentation**								
Stable angina	898 (36.7)	3592 (39.6)	0.010	0.059	545 (35.2)	659 (42.5)	<0.001	0.150
Unstable angina	1011 (41.3)	2624 (28.9)	<0.001	0.262	624 (40.3)	512 (33.0)	<0.001	0.152
NSTEMI	335 (13.7)	1608 (17.7)	<0.001	0.111	233 (15.0)	232 (15.0)	1.000	0.000
STEMI	203 (8.3)	1251 (13.8)	<0.001	0.176	145 (9.4)	146 (9.4)	1.000	0.000
**Treatment strategy**								
DCB-only treatment	1681 (68.7)	0	—	—	1077 (69.6)	0	—	—
**Target lesion**								
Left main	306 (12.5)	449 (4.9)	<0.001	0.270	147 (9.5)	146 (9.4)	0.990	0.001
LAD	1473 (60.2)	5445 (60.0)	0.879	0.004	931 (60.2)	930 (60.1)	0.987	0.001
LCX	1075 (43.9)	2704 (29.8)	<0.001	0.296	627 (40.5)	627 (40.5)	1.000	0.000
RCA	846 (34.6)	3500 (38.6)	<0.001	0.083	572 (37.0)	572 (37.0)	1.000	0.000
Multivessel PCI	972 (39.7)	2548 (28.1)	<0.001	0.248	571 (36.9)	617 (39.9)	0.018	0.061
**Disease extent**								
1-vessel disease	832 (34.0)	5456 (60.1)	<0.001	0.542	630 (40.7)	631 (40.8)	1.000	0.000
2-vessel disease	900 (36.8)	2431 (26.8)	<0.001	0.216	514 (33.2)	630 (40.7)	<0.001	0.156
3-vessel disease	715 (29.2)	1188 (13.1)	<0.001	0.403	403 (26.0)	287 (18.5)	<0.001	0.181
**Lesion complexity**								
Multivessel disease	1615 (66.0)	3619 (39.9)	<0.001	0.542	917 (59.3)	917 (59.2)	1.000	0.000
Bifurcation lesion	959 (39.2)	1090 (12.0)	<0.001	0.655	446 (28.9)	448 (28.9)	0.979	0.001
Chronic total occlusion	274 (11.2)	664 (7.3)	<0.001	0.134	155 (10.0)	155 (10.0)	1.000	0.000
ACC/AHA B2/C lesion	1770 (72.3)	4794 (52.8)	<0.001	0.412	1062 (68.6)	1062 (68.6)	1.000	0.000
**Vessel/device counts**								
Total number of devices used	1.8 ± 1.0	1.6 ± 0.8	<0.001	0.221	1.8 ± 1.0	1.8 ± 0.9	0.661	0.011

Total device length (mm)	46.5 ± 29.2	36.5 ± 22.6	<0.001	0.380	45.2 ± 28.8	40.4 ± 24.5	<0.001	0.180
Mean device diameter (mm)	2.8 ± 0.4	3.0 ± 0.4	<0.001	0.463	2.9 ± 0.4	2.9 ± 0.4	0.980	0.001
**DCB device characteristics**								
Total number of DCB used	1.4 ± 0.8	—	—	—	1.4 ± 0.7	—	—	—
Total DCB length (mm)	34.9 ± 22.0	—	—	—	34.0 ± 21.3	—	—	—
Mean DCB diameter (mm)	2.7 ± 0.4	—	—	—	2.8 ± 0.4	—	—	—
Small DCB used (diameter ≤2.5 mm)	1189 (48.6)	—	—	—	702 (45.3)	—	—	—
**DES device characteristics**								
Total number of DES used	0.4 ± 0.7	1.6 ± 0.8	<0.001	1.550	0.4 ± 0.7	1.8 ± 0.9	<0.001	1.686
Total DES length (mm)	11.6 ± 21.4	36.5 ± 22.6	<0.001	1.131	11.3 ± 21.2	40.4 ± 24.5	<0.001	1.275
Mean DES diameter (mm)	3.2 ± 0.5	3.0 ± 0.4	<0.001	0.315	3.2 ± 0.5	2.9 ± 0.4	<0.001	0.771
Small DES used (diameter ≤2.5 mm)	70 (9.1)	2170 (23.9)	<0.001	0.406	38 (8.1)	544 (35.2)	<0.001	0.696
**Follow-up**								
Follow-up duration (days)	1238.6 ± 943.9	1091.0 ± 969.8	<0.001	0.154	1236.8 ± 945.6	1218.2 ± 986.4	0.444	0.019

Values are mean ± SD or n (%). Unweighted columns show raw values with *p* values from Student's t-test (continuous) or chi-square test (categorical). Weighted columns show effective counts (sum of weights × proportion) with weighted statistics. SMD = standardized mean difference (absolute value).

Overlap weighting preserves the full original sample (n = 11,522); the reported counts and percentages in the overlap-weighted columns reflect reweighted distributions, not exclusion of any patients. Overlap weights here were derived from a 22-covariate propensity score model fitted to the entire cohort (after exclusion of two collinear procedural variables — total number of devices used and total device length; VIF > 5 — from the candidate 24-covariate set). Included covariates were age, male sex, hypertension, diabetes mellitus, current smoking, previous MI, previous PCI, previous stroke, left ventricular ejection fraction, hemoglobin, serum creatinine, left main disease, LAD, LCX, RCA, multivessel disease, bifurcation lesion, chronic total occlusion, ACC/AHA B2/C lesion, mean device diameter, and dummies for NSTEMI and STEMI presentation (unstable angina as reference). Stable angina was not separately indicated in this overall propensity score model (unstable angina served as the reference category with NSTEMI and STEMI indicators); consequently, the stable angina–acute coronary syndrome distribution is not balanced by the overall model, and presentation-defined inference relies on the predefined within-stratum analyses (Tables 2 and 3). Antiplatelet agents (clopidogrel, ticagrelor, prasugrel) are not mutually exclusive; patients who switched $P2Y_{12}$ inhibitors are counted under each agent received, so percentages may sum to >100%. Small DES used is the proportion among patients who received a drug-eluting stent

(hybrid procedures in the DCB-based group; all patients in the DES-only group).

ACC, American College of Cardiology; ACEI, angiotensin-converting enzyme inhibitor; ACS, acute coronary syndrome; AHA, American Heart Association; ARB, angiotensin receptor blocker; DAPT, dual antiplatelet therapy; DCB, drug-coated balloon; DES, drug-eluting stent; LAD, left anterior descending artery; LCX, left circumflex artery; LVEF, left ventricular ejection fraction; MI, myocardial infarction; NSTEMI, non–ST-segment elevation myocardial infarction; PCI, percutaneous coronary intervention; RCA, right coronary artery; SA, stable angina; SMD, standardized mean difference; STEMI, ST-segment elevation myocardial infarction; VIF, variance inflation factor.

Table S7 Baseline Characteristics within the DCB-based Group: Stable Angina versus Acute Coronary Syndrome

Variable	Unweighted (n = 2447)				Overlap-Weighted			
	SA (n = 898)	ACS (n = 1549)	*p* value	SMD	SA	ACS	*p* value	SMD
**Demographics and medical history**								
Age (years)	64.3 ± 9.7	63.6 ± 11.7	0.106	0.066	64.2 ± 9.7	64.2 ± 11.3	0.995	0.000
Male sex	708 (78.8)	1160 (74.9)	0.030	0.094	412 (76.9)	412 (76.9)	1.000	0.000
Hypertension	649 (72.3)	1016 (65.6)	<0.001	0.145	377 (70.2)	377 (70.2)	1.000	0.000
Diabetes mellitus	437 (48.7)	629 (40.6)	<0.001	0.163	246 (45.8)	246 (45.8)	1.000	0.000
Current smoking	207 (23.1)	470 (30.3)	<0.001	0.165	136 (25.4)	136 (25.4)	1.000	0.000
Previous MI	103 (11.5)	210 (13.6)	0.154	0.063	67 (12.4)	67 (12.4)	1.000	0.000
Previous PCI	234 (26.1)	424 (27.4)	0.510	0.030	144 (26.9)	144 (26.9)	1.000	0.001
Previous stroke	126 (14.0)	151 (9.7)	0.002	0.133	63 (11.8)	63 (11.8)	1.000	0.000
**Laboratory parameters**								
LVEF (%)	59.3 ± 9.5	56.6 ± 10.9	<0.001	0.258	58.4 ± 10.1	58.4 ± 9.6	0.997	0.000
Hemoglobin (g/dL)	13.6 ± 2.0	13.7 ± 2.0	0.140	0.062	13.6 ± 2.0	13.6 ± 2.0	0.995	0.000
Serum creatinine (mg/dL)	1.4 ± 1.9	1.3 ± 1.5	0.140	0.064	1.3 ± 1.8	1.3 ± 1.6	0.999	0.000
**Discharge medications**								
Aspirin	895 (99.7)	1545 (99.7)	1.000	0.014	535 (99.7)	536 (99.9)	0.736	0.034
Clopidogrel	889 (99.0)	1467 (94.7)	<0.001	0.248	531 (99.1)	511 (95.3)	<0.001	0.230
Ticagrelor	59 (6.6)	346 (22.3)	<0.001	0.460	38 (7.2)	108 (20.2)	<0.001	0.386
Prasugrel	9 (1.0)	59 (3.8)	<0.001	0.184	6 (1.0)	19 (3.6)	<0.001	0.170
DAPT duration (days)	637.0 ± 902.9	753.6 ± 961.4	0.003	0.125	634.1 ± 893.1	762.9 ± 980.9	0.001	0.137
Beta-blocker	377 (42.0)	912 (58.9)	<0.001	0.342	229 (42.8)	305 (56.9)	<0.001	0.285
ACEI or ARB	449 (50.0)	783 (50.5)	0.826	0.011	265 (49.4)	270 (50.4)	0.674	0.020

Statin	875 (97.5)	1506 (97.2)	0.727	0.020	524 (97.7)	521 (97.2)	0.508	0.035
**Clinical presentation**								
Stable angina	898 (100.0)	0	<0.001	—	536 (100.0)	0	<0.001	—
Unstable angina	0	1011 (65.3)	<0.001	1.939	0	374 (69.7)	<0.001	2.146
NSTEMI	0	335 (21.6)	<0.001	0.743	0	106 (19.7)	<0.001	0.701
STEMI	0	203 (13.1)	<0.001	0.549	0	56 (10.5)	<0.001	0.485
**Treatment strategy**								
DCB-only treatment	675 (75.2)	1006 (64.9)	<0.001	0.225	394 (73.5)	365 (68.1)	0.007	0.120
**Target lesion**								
Left main	93 (10.4)	213 (13.8)	0.017	0.104	61 (11.3)	61 (11.3)	1.000	0.000
LAD	527 (58.7)	946 (61.1)	0.263	0.049	320 (59.7)	320 (59.7)	1.000	0.000
LCX	377 (42.0)	698 (45.1)	0.151	0.062	230 (42.9)	230 (42.9)	1.000	0.000
RCA	289 (32.2)	557 (36.0)	0.064	0.080	179 (33.4)	179 (33.4)	1.000	0.000
Multivessel PCI	305 (34.0)	667 (43.1)	<0.001	0.188	197 (36.7)	200 (37.3)	0.805	0.013
**Disease extent**								
1-vessel disease	340 (37.9)	492 (31.8)	0.002	0.128	191 (35.6)	191 (35.6)	1.000	0.000
2-vessel disease	325 (36.2)	575 (37.1)	0.677	0.019	196 (36.6)	191 (35.6)	0.667	0.020
3-vessel disease	233 (25.9)	482 (31.1)	0.008	0.115	149 (27.7)	154 (28.7)	0.649	0.022
Lesion complexity								
Multivessel disease	558 (62.1)	1057 (68.2)	0.002	0.128	345 (64.4)	345 (64.4)	1.000	0.000
Bifurcation lesion	342 (38.1)	617 (39.8)	0.418	0.036	207 (38.6)	207 (38.6)	1.000	0.000
Chronic total occlusion	116 (12.9)	158 (10.2)	0.047	0.085	63 (11.7)	63 (11.7)	1.000	0.000
ACC/AHA B2/C lesion	602 (67.0)	1168 (75.4)	<0.001	0.186	377 (70.3)	377 (70.3)	1.000	0.000
**Vessel/device counts**								
Total number of devices used	1.8 ± 1.0	1.9 ± 1.0	0.015	0.102	1.8 ± 1.0	1.8 ± 1.0	0.611	0.022

Total device length (mm)	44.6 ± 28.0	47.5 ± 29.8	0.017	0.099	45.9 ± 28.7	45.2 ± 28.7	0.570	0.025
Mean device diameter (mm)	2.8 ± 0.4	2.8 ± 0.4	0.004	0.119	2.8 ± 0.4	2.8 ± 0.4	0.992	0.000
**DCB device characteristics**								
Total number of DCB used	1.4 ± 0.8	1.4 ± 0.7	0.104	0.069	1.4 ± 0.8	1.3 ± 0.7	0.005	0.124
Total DCB length (mm)	35.6 ± 22.7	34.4 ± 21.6	0.196	0.055	36.2 ± 23.2	33.6 ± 20.8	0.008	0.117
Mean DCB diameter (mm)	2.7 ± 0.4	2.7 ± 0.4	0.256	0.047	2.7 ± 0.4	2.7 ± 0.4	0.562	0.025
Small DCB used (diameter ≤2.5 mm)	431 (48.0)	758 (48.9)	0.685	0.019	252 (46.9)	274 (51.0)	0.065	0.081
**DES device characteristics**								
Total number of DES used	0.3 ± 0.6	0.5 ± 0.8	<0.001	0.211	0.4 ± 0.7	0.4 ± 0.7	0.021	0.099
Total DES length (mm)	9.0 ± 18.3	13.1 ± 22.8	<0.001	0.199	9.8 ± 19.0	11.7 ± 21.6	0.028	0.094
Mean DES diameter (mm)	3.1 ± 0.4	3.2 ± 0.5	0.417	0.064	3.1 ± 0.4	3.1 ± 0.5	0.828	0.018
Small DES used (diameter ≤2.5 mm)	21 (9.5)	49 (9.1)	0.980	0.013	13 (8.9)	17 (10.3)	0.660	0.048
**Follow-up**								
Follow-up duration (days)	1123.7 ± 899.8	1305.3 ± 962.3	<0.001	0.195	1115.1 ± 889.1	1314.7 ± 960.0	<0.001	0.216

Values are mean ± SD or n (%). Unweighted columns show raw values with *p* values from Student's t-test (continuous) or chi-square test (categorical). Weighted columns show effective counts (sum of weights × proportion) with weighted statistics. SMD = standardized mean difference (absolute value).

Overlap weighting preserves the full original sample (n = 2447 in the DCB-based group); the reported counts and percentages in the overlap-weighted columns reflect reweighted distributions, not exclusion of any patients. Antiplatelet agents (clopidogrel, ticagrelor, prasugrel) are not mutually exclusive; patients who switched P2Y12 inhibitors are counted under each agent received, so percentages may sum to >100%. Overlap weights were derived from a presentation-propensity score model fitted within the DCB-based group to compare SA with ACS. The modeled covariates were age, male sex, hypertension, diabetes mellitus, current smoking, previous MI, previous PCI, previous stroke, LVEF, hemoglobin, serum creatinine, left main lesion, LAD, LCX, RCA, multivessel disease, bifurcation lesion, chronic total occlusion, ACC/AHA B2/C lesion, and mean device diameter. Because discharge medications and device-specific variables were not included in this presentation-propensity model, their weighted differences should be interpreted descriptively.

ACC, American College of Cardiology; ACEI, angiotensin-converting enzyme inhibitor; ACS, acute coronary syndrome; AHA, American Heart Association; ARB, angiotensin receptor blocker; DAPT, dual antiplatelet therapy; DCB, drug-coated balloon; DES, drug-eluting stent; LAD, left anterior descending artery; LCX, left circumflex artery; LVEF, left ventricular ejection fraction; MI, myocardial infarction; NSTEMI, non–ST-segment elevation myocardial

infarction; PCI, percutaneous coronary intervention; RCA, right coronary artery; SA, stable angina; SMD, standardized mean difference; STEMI, ST-segment elevation myocardial infarction.

Table S8 Baseline Characteristics within the DES-only Group: Stable Angina versus Acute Coronary Syndrome

Variable	Unweighted (n = 9075)				Overlap-Weighted			
	SA (n = 3592)	ACS (n = 5483)	*p* value	SMD	SA	ACS	*p* value	SMD
**Demographics and medical history**								
Age (years)	64.6 ± 10.3	64.6 ± 11.5	0.890	0.003	64.7 ± 10.4	64.7 ± 11.3	0.999	0.000
Male sex	2456 (68.4)	3573 (65.2)	0.002	0.068	1372 (67.1)	1372 (67.1)	1.000	0.000
Hypertension	2279 (63.4)	3205 (58.5)	<0.001	0.102	1259 (61.6)	1260 (61.6)	1.000	0.000
Diabetes mellitus	1316 (36.6)	1766 (32.2)	<0.001	0.093	712 (34.8)	712 (34.8)	1.000	0.000
Current smoking	823 (22.9)	1789 (32.6)	<0.001	0.218	539 (26.3)	539 (26.3)	1.000	0.000
Previous MI	254 (7.1)	395 (7.2)	0.843	0.005	147 (7.2)	148 (7.2)	1.000	0.001
Previous PCI	462 (12.9)	725 (13.2)	0.641	0.011	270 (13.2)	270 (13.2)	1.000	0.000
Previous stroke	287 (8.0)	385 (7.0)	0.093	0.037	155 (7.6)	154 (7.6)	1.000	0.000
**Laboratory parameters**								
LVEF (%)	60.8 ± 10.1	57.5 ± 10.7	<0.001	0.315	59.6 ± 10.7	59.6 ± 9.9	0.998	0.000
Hemoglobin (g/dL)	13.5 ± 1.8	13.6 ± 1.9	0.475	0.015	13.5 ± 1.8	13.5 ± 1.9	0.999	0.000
Serum creatinine (mg/dL)	1.0 ± 1.0	1.1 ± 1.1	0.019	0.050	1.1 ± 1.1	1.1 ± 1.0	0.999	0.000
**Discharge medications**								
Aspirin	3470 (96.6)	5359 (97.7)	0.001	0.068	1978 (96.7)	1996 (97.6)	0.021	0.052
Clopidogrel	3592 (100.0)	5483 (100.0)	—	0.000	2045 (100.0)	2046 (100.0)	—	0.000
Ticagrelor	0	0	—	—	0	0	—	—
Prasugrel	0	0	—	—	0	0	—	—
DAPT duration (days)	625.7 ± 650.9	739.2 ± 715.5	<0.001	0.166	644.4 ± 667.2	705.1 ± 691.2	<0.001	0.089
Beta-blocker	1822 (50.7)	3526 (64.3)	<0.001	0.277	1038 (50.7)	1283 (62.7)	<0.001	0.243
ACEI or ARB	2147 (59.8)	3372 (61.5)	0.104	0.035	1224 (59.8)	1241 (60.7)	0.462	0.017

Statin	3187 (88.7)	4791 (87.4)	0.059	0.042	1815 (88.7)	1788 (87.4)	0.075	0.040
**Clinical presentation**								
Stable angina	3592 (100.0)	0	<0.001	—	2045 (100.0)	0	<0.001	—
Unstable angina	0	2624 (47.9)	<0.001	1.355	0	1089 (53.2)	<0.001	1.508
NSTEMI	0	1608 (29.3)	<0.001	0.911	0	562 (27.5)	<0.001	0.871
STEMI	0	1251 (22.8)	<0.001	0.769	0	394 (19.3)	<0.001	0.691
**Treatment strategy**								
DCB-only treatment	0	0	—	—	0	0	—	—
**Target lesion**								
Left main	195 (5.4)	254 (4.6)	0.097	0.036	105 (5.1)	105 (5.1)	1.000	0.000
LAD	2183 (60.8)	3262 (59.5)	0.232	0.026	1235 (60.4)	1235 (60.4)	1.000	0.000
LCX	1010 (28.1)	1694 (30.9)	0.005	0.061	600 (29.3)	600 (29.3)	1.000	0.000
RCA	1288 (35.9)	2212 (40.3)	<0.001	0.092	772 (37.7)	772 (37.7)	1.000	0.000
Multivessel PCI	932 (25.9)	1616 (29.5)	<0.001	0.079	574 (28.1)	549 (26.9)	0.230	0.027
**Disease extent**								
1-vessel disease	2320 (64.6)	3136 (57.2)	<0.001	0.152	1260 (61.6)	1260 (61.6)	1.000	0.000
2-vessel disease	910 (25.3)	1521 (27.7)	0.012	0.055	560 (27.4)	509 (24.9)	0.010	0.058
3-vessel disease	362 (10.1)	826 (15.1)	<0.001	0.151	225 (11.0)	277 (13.5)	<0.001	0.077
**Lesion complexity**								
Multivessel disease	1272 (35.4)	2347 (42.8)	<0.001	0.152	785 (38.4)	785 (38.4)	1.000	0.000
Bifurcation lesion	395 (11.0)	695 (12.7)	0.018	0.052	241 (11.8)	241 (11.8)	1.000	0.000
Chronic total occlusion	294 (8.2)	370 (6.7)	0.011	0.055	156 (7.6)	155 (7.6)	1.000	0.001
ACC/AHA B2/C lesion	1647 (45.9)	3147 (57.4)	<0.001	0.233	1032 (50.4)	1032 (50.4)	1.000	0.000
**Vessel/device counts**								
Total number of devices used	1.6 ± 0.8	1.6 ± 0.8	0.824	0.005	1.6 ± 0.8	1.6 ± 0.8	0.002	0.070

Total device length (mm)	37.1 ± 23.1	36.1 ± 22.3	0.052	0.042	37.8 ± 23.5	35.2 ± 21.9	<0.001	0.114
Mean device diameter (mm)	3.0 ± 0.4	3.0 ± 0.5	0.173	0.029	3.0 ± 0.4	3.0 ± 0.4	0.993	0.000
**DCB device characteristics**								
Total number of DCB used	—	—	—	—	—	—	—	—
Total DCB length (mm)	—	—	—	—	—	—	—	—
Mean DCB diameter (mm)	—	—	—	—	—	—	—	—
Small DCB used (diameter ≤2.5 mm)	—	—	—	—	—	—	—	—
**DES device characteristics**								
Total number of DES used	1.6 ± 0.8	1.6 ± 0.8	0.824	0.005	1.6 ± 0.8	1.6 ± 0.8	0.002	0.070
Total DES length (mm)	37.1 ± 23.1	36.1 ± 22.3	0.052	0.042	37.8 ± 23.5	35.2 ± 21.9	<0.001	0.114
Mean DES diameter (mm)	3.0 ± 0.4	3.0 ± 0.5	0.173	0.029	3.0 ± 0.4	3.0 ± 0.4	0.993	0.000
Small DES used (diameter ≤2.5 mm)	865 (24.1)	1305 (23.8)	0.779	0.007	492 (24.1)	495 (24.2)	0.896	0.003
**Follow-up**								
Follow-up duration (days)	967.5 ± 920.6	1171.9 ± 992.4	<0.001	0.213	997.0 ± 928.6	1125.4 ± 984.5	<0.001	0.134

Values are mean ± SD or n (%). Unweighted columns show raw values with *p* values from Student's t-test (continuous) or chi-square test (categorical). Weighted columns show effective counts (sum of weights × proportion) with weighted statistics. SMD = standardized mean difference (absolute value).

Overlap weighting preserves the full original sample (n = 9075 in the DES-only group); the reported counts and percentages in the overlap-weighted columns reflect reweighted distributions, not exclusion of any patients. Overlap weights were derived from a presentation-propensity score model fitted within the DES-only group to compare SA with ACS. The modeled covariates were age, male sex, hypertension, diabetes mellitus, current smoking, previous MI, previous PCI, previous stroke, LVEF, hemoglobin, serum creatinine, left main lesion, LAD, LCX, RCA, multivessel disease, bifurcation lesion, chronic total occlusion, ACC/AHA B2/C lesion, and mean device diameter. Because discharge medications and device-specific variables were not included in this presentation-propensity model, their weighted differences should be interpreted descriptively.

ACC, American College of Cardiology; ACEI, angiotensin-converting enzyme inhibitor; ACS, acute coronary syndrome; AHA, American Heart Association; ARB, angiotensin receptor blocker; DAPT, dual antiplatelet therapy; DCB, drug-coated balloon; DES, drug-eluting stent; LAD, left anterior descending artery; LCX, left circumflex artery; LVEF, left ventricular ejection fraction; MI, myocardial infarction; NSTEMI, non–ST-segment elevation myocardial infarction; PCI, percutaneous coronary intervention; RCA, right coronary artery; SA, stable angina; SMD, standardized mean difference; STEMI, ST-segment elevation

myocardial infarction.

Table S9 Unadjusted and Adjusted Predictors of 3-Year MACCE by Clinical Presentation.

(A) Stable Angina (n = 4490; 315 MACCE events)

Variable	Unadjusted HR (95% CI)	*p* value	Adjusted HR (95% CI)	*p* value
DCB-based vs DES-only strategy	0.37 (0.26–0.53)	<0.001	0.33 (0.23–0.49)	<0.001
Age, per 1 year	1.01 (1.00–1.02)	0.035	1.01 (1.00–1.02)	0.087
Male sex	1.17 (0.91–1.50)	0.216	1.31 (1.00–1.72)	0.054
Hypertension	1.24 (0.97–1.57)	0.083	1.18 (0.92–1.51)	0.199
Diabetes mellitus	1.24 (1.00–1.55)	0.054	1.21 (0.96–1.52)	0.101
Current smoking	0.91 (0.70–1.19)	0.484	0.91 (0.68–1.20)	0.489
Previous MI	0.82 (0.52–1.27)	0.370	0.68 (0.41–1.10)	0.117
Previous PCI	1.10 (0.82–1.48)	0.515	1.31 (0.95–1.82)	0.103
Previous stroke	1.21 (0.85–1.72)	0.298	1.25 (0.87–1.79)	0.230
LVEF, per 1%	0.98 (0.97–0.99)	0.001	0.98 (0.97–0.99)	0.003
Hemoglobin, per 1 g/dL	0.97 (0.92–1.03)	0.316	1.01 (0.97–1.05)	0.663
Serum creatinine, per 1 mg/dL	1.11 (1.05–1.17)	<0.001	1.11 (1.04–1.18)	0.001
Left main lesion	1.09 (0.72–1.67)	0.677	1.59 (0.99–2.55)	0.055
LAD lesion	0.99 (0.79–1.25)	0.956	1.30 (0.96–1.76)	0.086
LCX lesion	1.00 (0.79–1.27)	0.990	1.25 (0.92–1.71)	0.157
RCA lesion	1.32 (1.05–1.65)	0.016	1.44 (1.06–1.96)	0.020
Multivessel disease	1.02 (0.82–1.28)	0.831	1.02 (0.72–1.43)	0.924
Bifurcation lesion	0.64 (0.46–0.88)	0.006	0.76 (0.53–1.08)	0.120
Chronic total occlusion	1.11 (0.78–1.60)	0.557	1.17 (0.81–1.70)	0.392
ACC/AHA type B2/C lesion	0.83 (0.67–1.04)	0.109	0.75 (0.57–0.99)	0.040

(B) Acute Coronary Syndrome (n = 7032; 597 MACCE events)

Variable	Unadjusted HR (95% CI)	*p* value	Adjusted HR (95% CI)	*p* value
DCB-based vs DES-only strategy	0.88 (0.73–1.07)	0.202	0.78 (0.63–0.97)	0.024
NSTEMI (vs unstable angina)	1.21 (1.02–1.44)	0.029	1.09 (0.89–1.32)	0.414
STEMI (vs unstable angina)	1.00 (0.82–1.21)	0.970	1.08 (0.85–1.36)	0.533
Age, per 1 year	1.02 (1.01–1.02)	<0.001	1.01 (1.00–1.02)	0.012
Male sex	1.07 (0.90–1.27)	0.433	1.23 (1.02–1.49)	0.031
Hypertension	1.42 (1.20–1.68)	<0.001	1.27 (1.05–1.52)	0.011

Diabetes mellitus	1.38 (1.17–1.62)	<0.001	1.16 (0.97–1.37)	0.098
Current smoking	0.94 (0.79–1.12)	0.499	1.08 (0.89–1.31)	0.431
Previous MI	1.38 (1.07–1.76)	0.012	0.96 (0.71–1.30)	0.800
Previous PCI	1.47 (1.21–1.78)	<0.001	1.34 (1.06–1.69)	0.016
Previous stroke	1.54 (1.19–1.99)	0.001	1.22 (0.93–1.58)	0.145
LVEF, per 1%	0.98 (0.97–0.98)	<0.001	0.98 (0.97–0.99)	<0.001
Hemoglobin, per 1 g/dL	0.90 (0.86–0.93)	<0.001	0.95 (0.91–1.00)	0.034
Serum creatinine, per 1 mg/dL	1.13 (1.08–1.17)	<0.001	1.06 (1.01–1.11)	0.024
Left main lesion	1.77 (1.38–2.28)	<0.001	1.97 (1.49–2.61)	<0.001
LAD lesion	1.03 (0.88–1.22)	0.700	1.11 (0.91–1.35)	0.289
LCX lesion	0.95 (0.80–1.12)	0.537	0.94 (0.76–1.16)	0.554
RCA lesion	1.15 (0.98–1.35)	0.088	1.10 (0.90–1.35)	0.342
Multivessel disease	1.21 (1.03–1.43)	0.020	1.17 (0.94–1.45)	0.167
Bifurcation lesion	0.95 (0.78–1.16)	0.594	0.85 (0.68–1.07)	0.176
Chronic total occlusion	1.26 (0.96–1.66)	0.102	1.20 (0.90–1.59)	0.205
ACC/AHA type B2/C lesion	0.93 (0.79–1.10)	0.417	0.79 (0.65–0.96)	0.019

Unadjusted hazard ratios were derived from univariable Cox proportional hazards models; adjusted hazard ratios were derived from a multivariable Cox model including all listed variables simultaneously. The DCB-based strategy variable contrasts a DCB-based with a DES-only approach (reference). In the acute coronary syndrome panel, NSTEMI and STEMI are compared with unstable angina (reference). Because this full predictor model characterizes all covariate associations simultaneously rather than being specified to isolate the treatment contrast, the DCB-based hazard ratio differs marginally from the treatment-effect multivariable Cox estimate in Tables S4 and S5.

ACC/AHA, American College of Cardiology/American Heart Association; ACS, acute coronary syndrome; CI, confidence interval; DCB, drug-coated balloon; DES, drug-eluting stent; HR, hazard ratio; LAD, left anterior descending coronary artery; LCX, left circumflex artery; LVEF, left ventricular ejection fraction; MACCE, major adverse cardiac and cerebrovascular events; MI, myocardial infarction; NSTEMI, non–ST-segment elevation myocardial infarction; PCI, percutaneous coronary intervention; RCA, right coronary artery; SA, stable angina; STEMI, ST-segment elevation myocardial infarction.

Table S10 Additional Sensitivity Analyses of 3-Year Outcomes: Composition of the DCB-based Strategy, Influence of STEMI, and Bleeding in Relation to DAPT Duration.

Analysis	DCB-based n (events)	DES-only n (events)	Overlap-weighted HR (95% CI)	p value
**A. Composition of the DCB-based strategy — 3-year MACCE**				
Stable angina				
Primary analysis (all DCB-based)	898 (33)	3592 (282)	0.33 (0.22–0.49)	<0.001
DCB-only PCI vs. DES-only	675 (21)	3592 (282)	0.30 (0.19–0.49)	<0.001
Hybrid DCB–DES PCI vs. DES-only	223 (12)	3592 (282)	0.43 (0.23–0.82)	0.011
Acute coronary syndrome				
Primary analysis (all DCB-based)	1549 (134)	5483 (463)	0.73 (0.59–0.91)	0.005
DCB-only PCI vs. DES-only	1006 (88)	5483 (463)	0.78 (0.60–1.01)	0.055
Hybrid DCB–DES PCI vs. DES-only	543 (46)	5483 (463)	0.71 (0.51–1.00)	0.051
**B. Influence of STEMI — 3-year MACCE, acute coronary syndrome stratum**				
Excluding STEMI (unstable angina and NSTEMI)	1346 (110)	4232 (362)	0.68 (0.53–0.86)	0.001
STEMI only	203 (24)	1251 (101)	0.97 (0.56–1.70)	0.927
**C. Major bleeding (BARC 3–5) and duration of DAPT**				
Stable angina				
Primary analysis	898 (8)	3592 (89)	0.32 (0.15–0.69)	0.003
Additionally adjusted for DAPT duration	891 (8)	3592 (89)	0.29 (0.14–0.63)	0.002
Acute coronary syndrome				
Primary analysis	1549 (19)	5483 (180)	0.32 (0.19–0.53)	<0.001
Additionally adjusted for DAPT duration	1540 (19)	5483 (180)	0.30 (0.18–0.51)	<0.001

All analyses used overlap weights derived from propensity score models fitted within the relevant stratum, with the same covariates as the principal analysis (20 in the stable angina stratum, 22 in the acute coronary syndrome stratum); analyses restricted to a subset of patients had the propensity score re-estimated within that subset, and the analysis confined to STEMI omitted the presentation indicators. Hazard ratios below 1.0 indicate fewer events with the DCB-based strategy; all models used robust variance estimators with follow-up censored at 3 years, and event counts are unweighted. DAPT duration was entered as a continuous covariate; because it is recorded after the treatment decision, the corresponding estimates are exploratory. BARC, Bleeding Academic Research Consortium; CI, confidence interval; DAPT, dual antiplatelet therapy; DCB, drug-coated balloon; DES, drug-eluting stent; HR, hazard ratio; MACCE, major adverse cardiac and cerebrovascular events; NSTEMI, non–ST-segment elevation myocardial infarction; PCI, percutaneous coronary intervention; STEMI, ST-segment elevation myocardial infarction.

Figure S1 Propensity score distribution and covariate balance in the SA cohort and in the ACS cohort

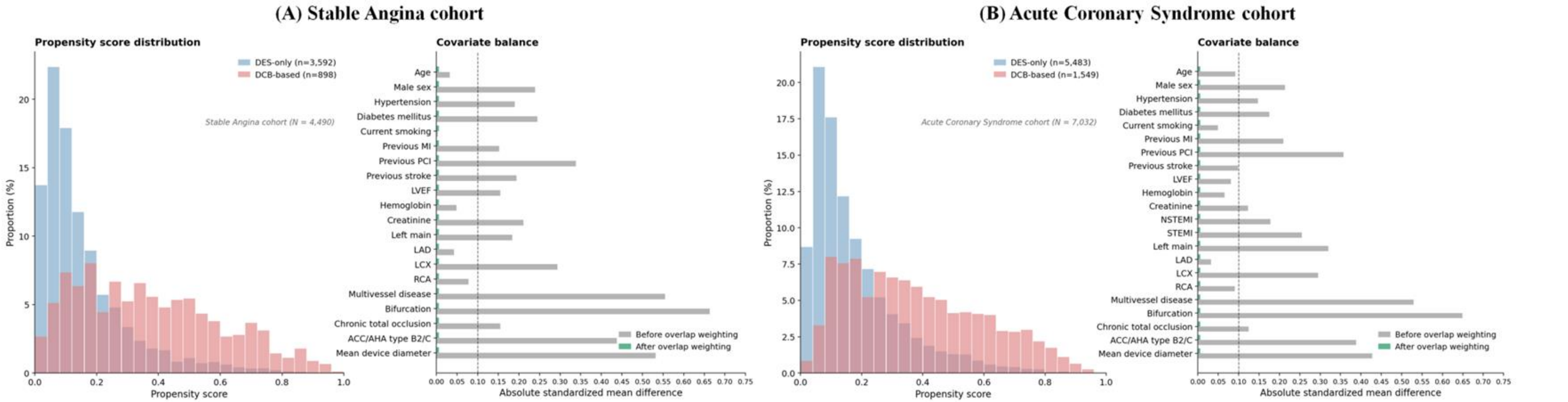

Panel A shows the Stable Angina cohort and Panel B shows the Acute Coronary Syndrome cohort. Within each panel, the left sub-panel displays the distribution of estimated propensity scores for assignment to the drug-coated balloon (DCB)–based strategy versus the drug-eluting stent (DES)–only strategy, plotted separately for each treatment group; the substantial overlap of the two distributions across the full range of propensity scores supports the positivity assumption required for causal inference. The right sub-panel displays absolute standardized mean differences (SMDs) for each baseline covariate included in the propensity score model—20 covariates in the Stable Angina stratum and 22 covariates in the Acute Coronary Syndrome stratum—before (gray bars) and after (green bars) overlap weighting. The vertical dashed line marks the conventional balance threshold of 0.1. After overlap weighting, all absolute SMDs were ≤ 0.001, well within the conventional threshold, with the green bars appearing uniformly minimal because overlap weighting achieves exact mean balance for every modeled covariate by design.

ACC, American College of Cardiology; AHA, American Heart Association; DCB, drug-coated balloon; DES, drug-eluting stent; LAD, left anterior descending artery; LCX, left circumflex artery; LVEF, left ventricular ejection fraction; MI, myocardial infarction; NSTEMI, non–ST-segment elevation myocardial infarction; PCI, percutaneous coronary intervention; RCA, right coronary artery; SMD, standardized mean difference; STEMI, ST-segment elevation myocardial infarction.

Figure S2 Subgroup analysis of DCB-based versus DES-only PCI for 3-year MACCE (overlap-weighted)

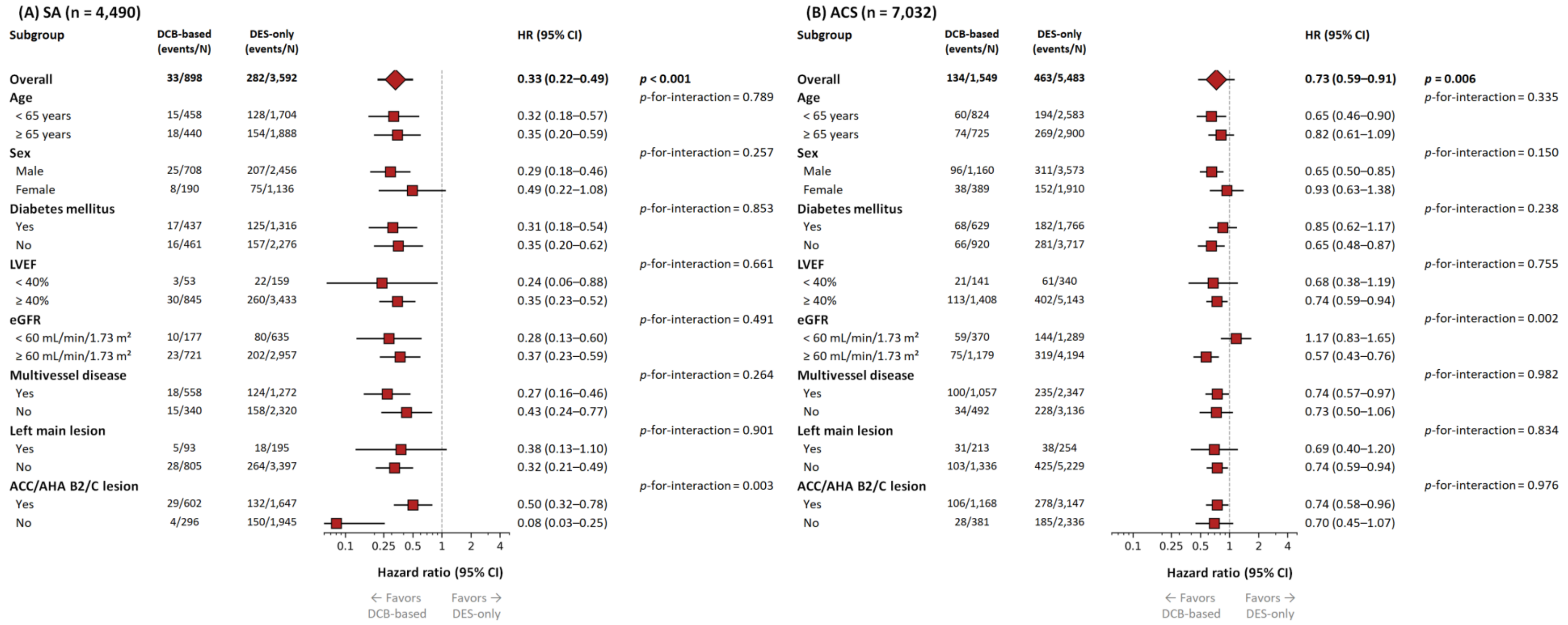

Forest plots show overlap-weighted hazard ratios (HRs) with 95% confidence intervals (CIs) for MACCE in predefined subgroups within the stable angina cohort (panel A; n = 4490) and the acute coronary syndrome cohort (panel B; n = 7032). The overall estimate for each cohort is shown at the top of each plot (red diamond), followed by subgroup-specific estimates (red squares with horizontal whiskers indicating the 95% CI). The vertical dashed line marks an HR of 1; an HR below 1 favors the DCB-based strategy. Event counts are shown as the number of MACCE events divided by the number of patients in each subgroup.

Within each subgroup, treatment effects were estimated by overlap-weighted Cox proportional-hazards models using the within-stratum propensity score weights; robust (sandwich) standard errors were used throughout. *P* values for interaction were obtained from overlap-weighted Cox models with a treatment-by-subgroup interaction term and assess whether the treatment effect differed across subgroup categories on the log-hazard scale.

ACC, American College of Cardiology; ACS, acute coronary syndrome; AHA, American Heart Association; CI, confidence interval; DCB, drug-coated balloon; DES, drug-eluting stent; eGFR, estimated glomerular filtration rate; HR, hazard ratio; LVEF, left ventricular ejection fraction; MACCE, major adverse cardiac and cerebrovascular events; PCI, percutaneous coronary intervention; SA, stable angina.

Figure S3 Three-year cumulative incidence of clinical outcomes that did not differ significantly between treatment strategies, stratified by clinical presentation

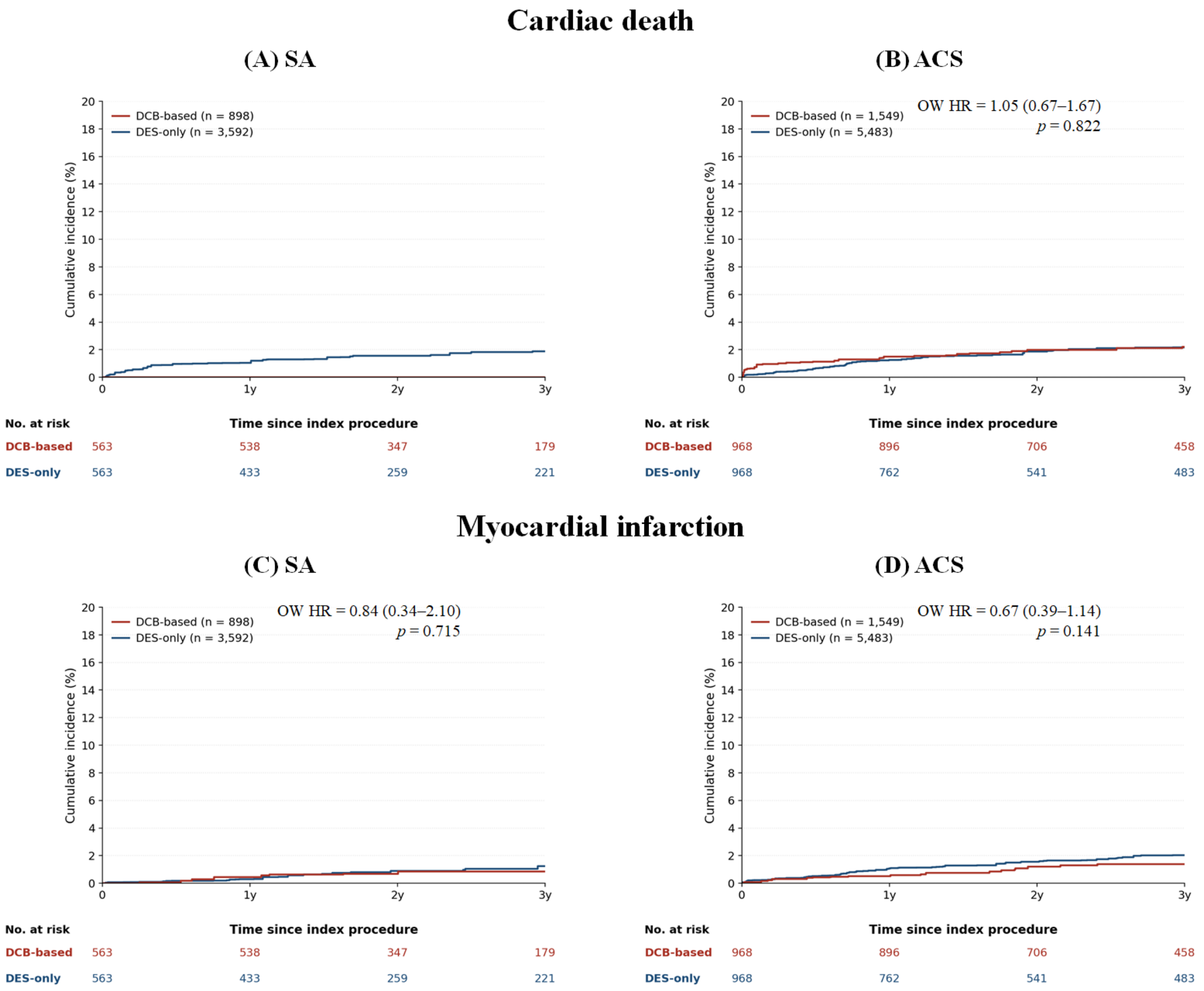

Stroke

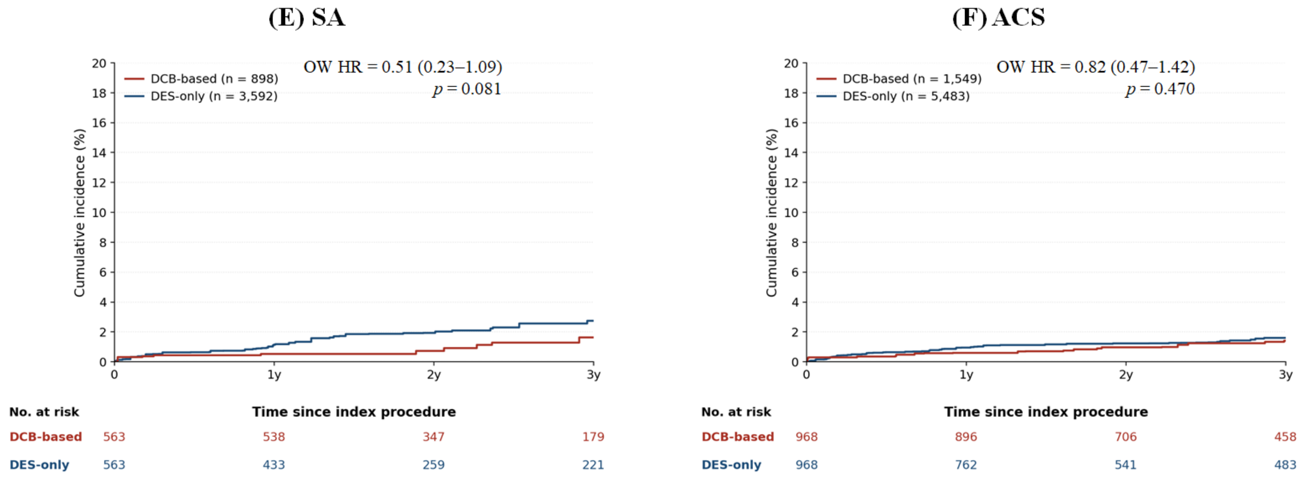

Each row presents one endpoint, with the stable angina (SA) stratum on the left and the acute coronary syndrome (ACS) stratum on the right: cardiac death (panels A and B), myocardial infarction (panels C and D), and stroke (panels E and F). Cumulative incidence was estimated by the Kaplan–Meier method after overlap weighting. Hazard ratios (HRs) with 95% confidence intervals (CIs) were obtained from overlap-weighted Cox proportional-hazards models with robust (sandwich) standard errors; an HR below 1 favors the DCB-based strategy. P values are derived from the same weighted models. Overlap weights were estimated separately within each clinical stratum from a propensity score model that included 20 covariates in the SA stratum and 22 covariates in the ACS stratum, the two strata differing by the inclusion of NSTEMI and STEMI indicators in the ACS model. Numbers below each panel are the overlap-weighted effective patients at risk at yearly intervals. For cardiac death in the SA stratum (panel A), no events occurred in the DCB-based group, so the hazard ratio and *p* value are not estimable and are omitted owing to monotone likelihood.

Figure S4 Three-year cumulative incidence of clinical outcomes comparing stable angina with acute coronary syndrome within each treatment group

MACCE

(A) DCB-based group

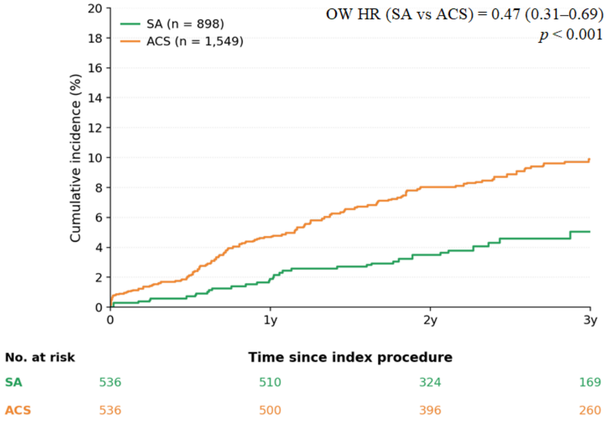

(B) DES-only group

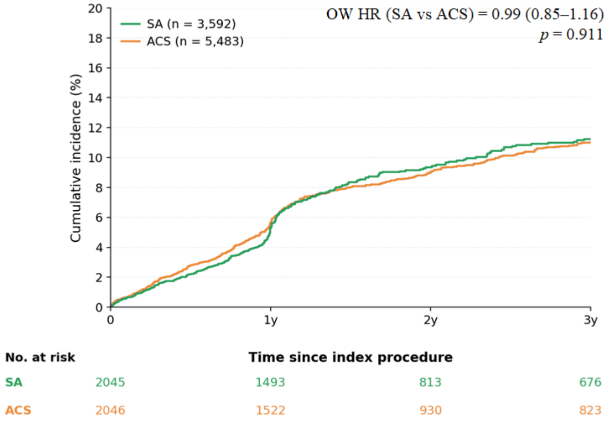

Cardiac death

(A) DCB-based group

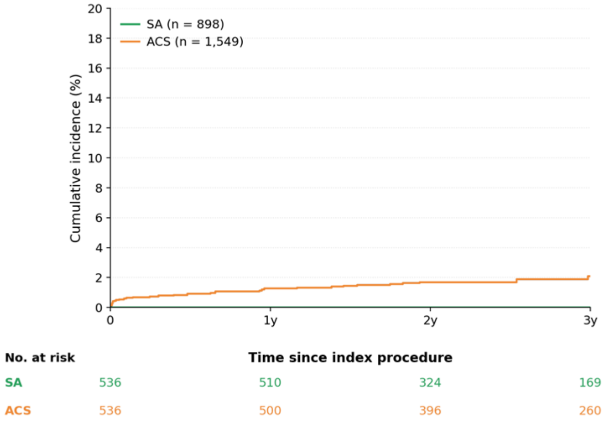

(B) DES-only group

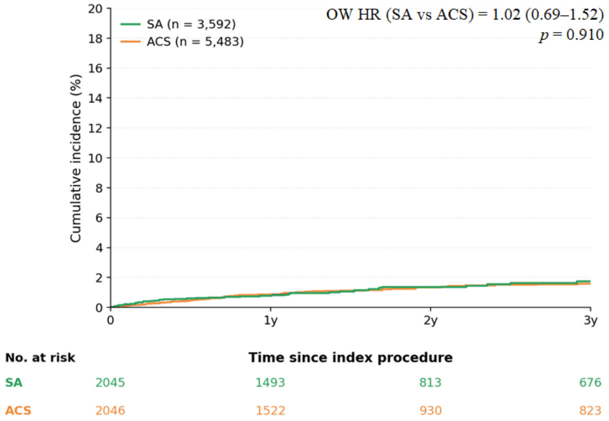

Myocardial infarction

(A) DCB-based group

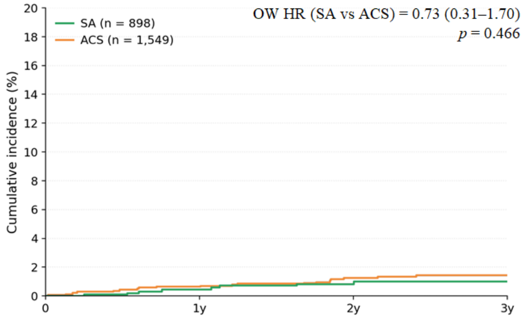

No. at risk	Time since index procedure			
SA	536	510	324	169
ACS	536	500	396	260

(B) DES-only group

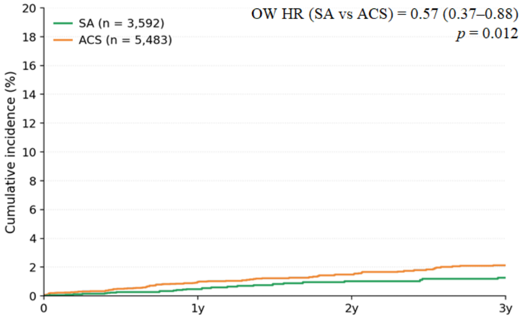

No. at risk	Time since index procedure			
SA	2045	1493	813	676
ACS	2046	1522	930	823

Target-vessel revascularization

(A) DCB-based group

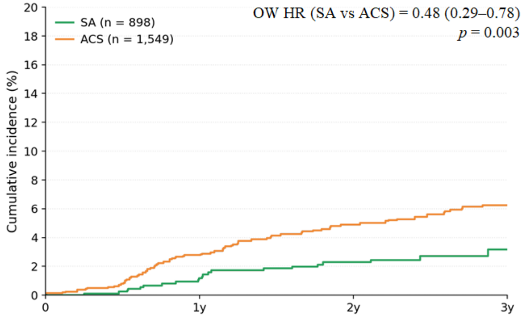

No. at risk	Time since index procedure			
SA	536	510	324	169
ACS	536	500	396	260

(B) DES-only group

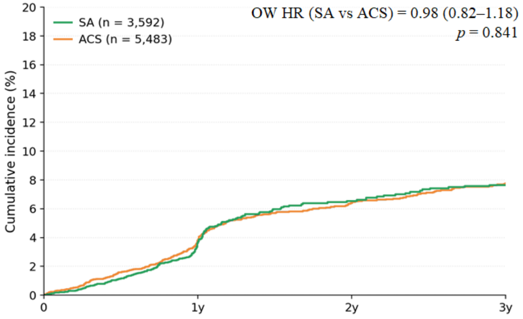

No. at risk	Time since index procedure			
SA	2045	1493	813	676
ACS	2046	1522	930	823

Target-lesion or stent thrombosis

(A) DCB-based group

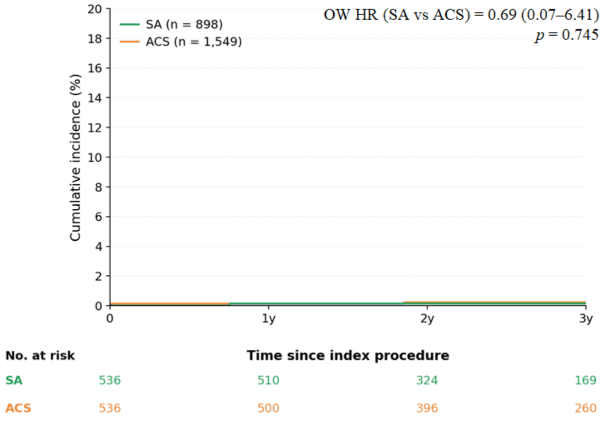

(B) DES-only group

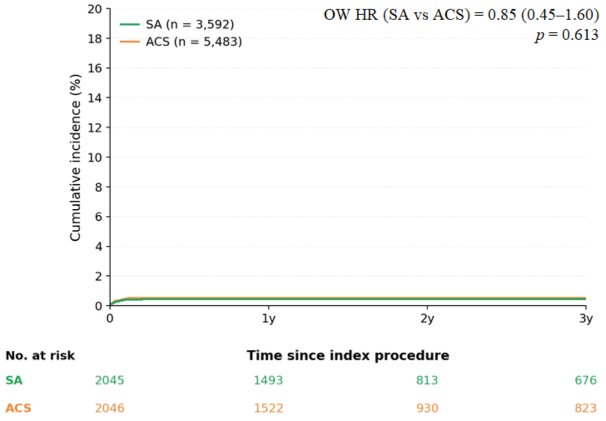

Stroke

(A) DCB-based group

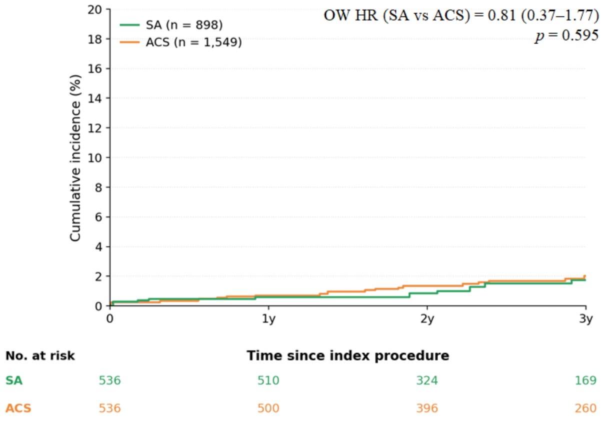

(B) DES-only group

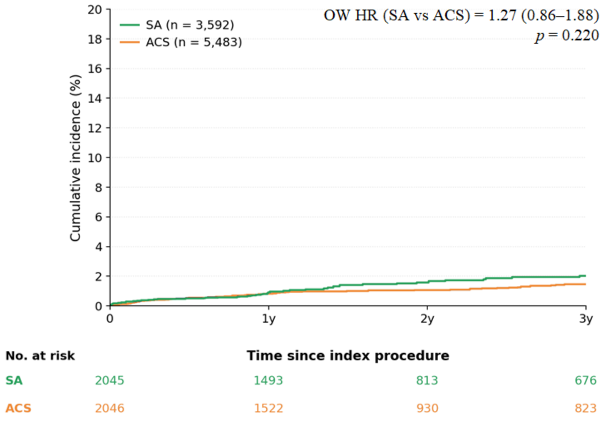

Major bleeding

(A) DCB-based group

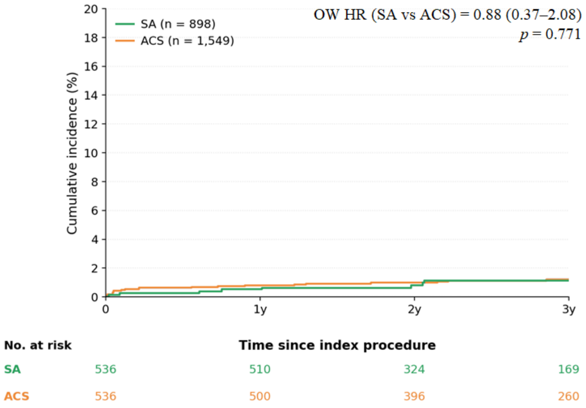

(B) DES-only group

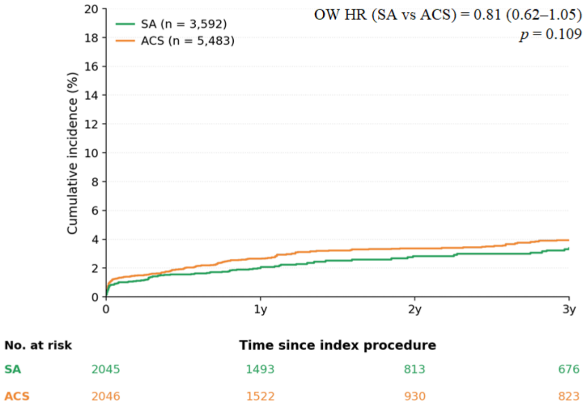

NACE

(A) DCB-based group

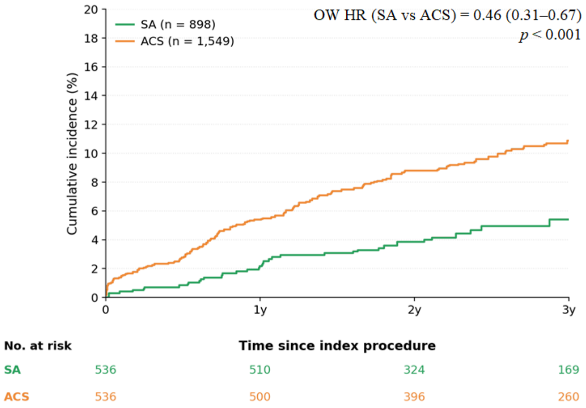

(B) DES-only group

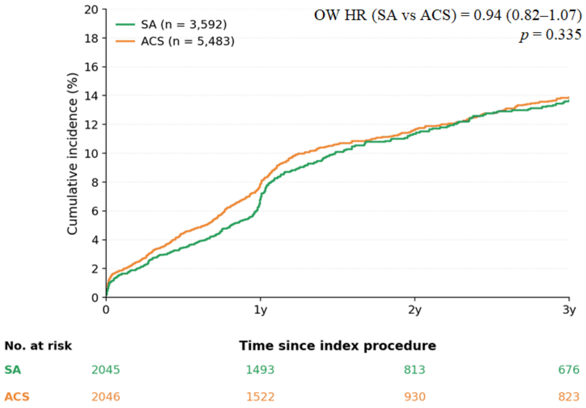

Each row presents one endpoint, with the drug-coated balloon (DCB)–based group on the left (panel A) and the drug-eluting stent (DES)–only group on the right (panel B). Endpoints are displayed in the same order as Table 4: major adverse cardiac and cerebrovascular events (MACCE), cardiac death, myocardial infarction, target-vessel revascularization, target-lesion or stent thrombosis, stroke, major bleeding, and net adverse clinical events (NACE).

Cumulative incidence was estimated by the Kaplan–Meier method after overlap weighting. Hazard ratios (HRs) with 95% confidence intervals (CIs) compare SA with ACS within each treatment group and were obtained from overlap-weighted Cox proportional-hazards models with robust (sandwich) standard errors; an HR below 1 indicates a lower event rate in SA than in ACS. *P* values are derived from the same weighted models. Overlap weights are the within-group propensity score weights used in Table 4, balancing SA and ACS within each treatment group. Numbers below each panel are the overlap-weighted effective patients at risk at yearly intervals.

Comparing the two panels within each row illustrates the basis of the treatment-by-presentation interaction reported in the main analyses: within the DCB-based group, SA patients had a markedly lower MACCE rate than ACS patients (HR 0.47, 95% CI 0.31–0.69), whereas within the DES-only group the two presentations had nearly identical event rates (HR 0.99, 95% CI 0.85–1.16). A similar pattern was observed for target-vessel revascularization and net adverse clinical events.

For cardiac death within the DCB-based group, no events occurred in SA patients, so the hazard ratio and P value are not estimable and are omitted owing to monotone likelihood.

ACS, acute coronary syndrome; CI, confidence interval; DCB, drug-coated balloon; DES, drug-eluting stent; HR, hazard ratio; MACCE, major adverse cardiac and cerebrovascular events; NACE, net adverse clinical events; OW, overlap weighting; SA, stable angina; TVR, target-vessel revascularization.
